# Crossing the Tethys on “biotic ferries”: new mayfly genera of Gondwanan origin in Mesozoic and Cenozoic deposits of Myanmar and India (Insecta: Ephemeroptera: Teloganodidae)

**DOI:** 10.1186/s12862-025-02488-3

**Published:** 2026-03-17

**Authors:** Roman J. Godunko, Corentin Jouault, Alexander V. Martynov, Zhendong Lian, Arnold H. Staniczek

**Affiliations:** 1 Biology Centre of the Czech Academy of Sciences, Institute of Entomology, Branišovská 31, České Budějovice, 37005 Czech Republic; 2 Department of Invertebrate Zoology and Hydrobiology, Faculty of Biology and Environmental Protection, University of Lodz, Banacha 12/16, Łódź, 90237 Poland; 3 State Museum of Natural History, National Academy of Sciences of Ukraine, Teatralna 18, Lviv, 79008 Ukraine; 4 Oxford University Museum of Natural History, University of Oxford, Parks Road, Oxford, OX1 3PW UK; 5 National Museum of Natural History, National Academy of Sciences of Ukraine, Bohdan Khmelnytsky 15, Kyiv, 01030 Ukraine; 6 2F., No. 138, Aly. 118, Ln. 235, Dedong St., East Dist., Tainan City, 701030 Taiwan, China; 7 Department of Entomology, State Museum of Natural History Stuttgart, Rosenstein 1, 70191 Stuttgart, Germany

**Keywords:** Ephemerelloidea, Pantricorythi, Burmese amber, Palana formation, Cretaceous, Late palaeocene, Early eocene, Burma Terrane, Indian plate

## Abstract

**Backgound:**

Mesozoic and Cenozoic fossils from Myanmar and the Indian subcontinent provide a crucial source of information on the global distribution of biota after the breakup of East Gondwana and the subsequent northward drift of these land masses towards Asia. While the mayfly fauna of Burmese amber is relatively well studied, data on extinct Ephemeroptera from India remain scarce.

**Results:**

Here, we describe for the first time a fossil adult mayfly reliably assigned to the family Teloganodidae. Based on a single male imago discovered in mid-Cretaceous Burmese amber, we establish a new species and genus, *Chibiphemera cretalota* **gen. & sp. nov.** based on the following combination of characters: (**i**) small body and forewing size; (**ii**) distinctive forewing venation, including the positions of RS and MP forks; (**iii**) strongly reduced hind wings, with a distally positioned costal process and markedly diminished venation; (**iv**) presence of pad-like pretarsal claws on the forelegs together with pointed claws on the middle and hind legs; and (**v**) unique shape of genitalia characterized by deeply separated, stick-like penis lobes and a markedly elongated gonostyli segment I. Adult morphological characters show affinities between the fossil *Chibiphemera* **gen. nov.** and extant South African and Malagasy genera of Teloganodidae. Plesiomorphies in the forewings and genitalia of *Chibiphemera* **gen. nov.** suggest an early diverged position within the family. Additionally, we re-examine the holotype of the Cenozoic species *Teloganella gurhaensis* Agnihotri et al., 2020, originally described from a single larva from the late Palaeocene–early Eocene Palana Formation (India), and redescribe it. It is defined by larval characters such as (**i**) a broad pronotum protruding anterolaterally, (**ii**) strongly expanded femora that are widest centrally, (**iii**) tibiae moderately widened distally, (**iv**) robust, hooked pretarsal claws, (**v**) a small, styliform gill I attached to abdominal segment I near its outer margin medially, and (**vi**) the presence of three caudal filaments. We establish a monotypic genus *Bharataganodes* **gen. nov.** for *Bharataganodes gurhaensis* (Agnihotri et al., 2020) **comb. nov.**, and discuss its systematic position within Teloganodidae and its relationships with other genera.

**Conclusions:**

Together, these fossils support a Gondwanan origin for Teloganodidae and highlight the importance of the Burma Terrane and the Indian Plate as key dispersal routes for aquatic insects across the Tethys to Asia during the Mesozoic–Cenozoic transition.

## Background

The evolutionary history of many mayfly lineages remains fragmentarily studied due to the small number and poor preservation of their fossil remains [1]. While paleolake deposits contain a rich and relatively well-preserved fauna documented by larvae and adults [2–7], presumably rheophilic groups of Ephemeroptera are very rarely preserved as compression fossils due to taphonomic constraints in lotic paleoecosystems [1, 8, 9]. Likewise rare and unique are the finds of small-sized mayflies, which are seldom preserved as compressed fossils in a condition suitable for the precise establishment of their systematic position. In this case, fossil resins from the Mesozoic and Cenozoic provide a more reliable source of such information [10–14].

Ephemeroptera are an ancient, ecologically relatively conservative order. Most species are stenobionts, their nymphs preferring a narrow range of freshwater ecological conditions. Their short-lived and relatively fragile adult stages also have a limited potential for dispersal. As a result, they are often confined to a narrow distributional range [15–17]. These ecological traits make mayflies excellent subjects for historical biogeographical studies and generalized scenarios, particularly in reconstructing distribution patterns shaped by continental drift and fragmentation, alongside other freshwater groups that cannot disperse by using ocean currents [17, 18].

Teloganodidae Allen, 1965, a small mayfly family with extant representatives in South Africa, Madagascar, and the Oriental Realm, is widely regarded as a Gondwanan relict lineage, whose distribution reflects tectonic vicariance following the breakup of Gondwana [17, 19–25]. Teloganodidae in the Oriental Realm are represented by four extant genera, i.e. the most diverse *Dudgeodes* Sartori, 2008 and *Teloganodes* Eaton, 1882, as well as *Derlethina* Sartori, 2008 with two described species from Borneo and India, and *Indoganodes* Selvakumar et al., 2014 with two species described from India and Sri Lanka [17, 26]. Some authors [27, 28] suggested a genus *Teloganodes sensu lato*, including three respective subgenera (see Table 1 for summarised information about the taxonomical composition of Teloganodidae). The extant African and Madagascar lineages of Teloganodidae are well studied ([17; see also Table 1) . The distribution of the African genera is restricted to the Cape Province of the Republic of South Africa (RSA). *Ephemerellina* Lestage, 1924 and *Lestagella* Demoulin, 1970 are both monospecific, while the genus *Nadinetella* McCafferty & Wang, 1998 is reported with two species (for more details see [17]). Finally, *Manohyphella* Allen, 1973 is endemic to Madagascar (Table 1).

**Table 1:** Overview of the Teloganodidae and Teloganellidae diversity and distribution. Extinct taxa are marked with “†”. Only described species are indicated under mentioned genera

Genera	Number of the species	Distribution	Principal references
**Indomalayan Realm**			
†*Bharataganodes * **gen. nov.**^A^	1	India: Palana Formation, Rajasthan; Late Paleocene–early Eocene	[29] and this contribution
*Dudgeodes* Sartori, 2008	17	Bali, Borneo, Java, Sulawesi, Sumatra, China, India, Philippines, Thailand	[24, 27, 28]^B^
*Derlethina* Sartori, 2008	2	Borneo, India	[24, 25, 27, 28]^B^
*Teloganella* Ulmer, 1939 = *Janohyphella* Selvakumar, Sivaramakrishnan & Jacobus, 2014	2	Sumatra, India, Malaysia	[30–32]
*Teloganodes* Eaton, 1882 = *Macafertiella* Wang, 1996	6^C, D^	Java, Sumatra, China, India, Philippines, Sri Lanka, Vietnam	[24, 27, 28, 33]
*Indoganodes* Selvakumar, Sivaramakrishnan & Jacobus, 2014	2	India, Sri Lanka	[25, 26, 34]
†*Chibiphemera * **gen. nov.**	1	Myanmar: Kachin State, Hukawng Valley; mid-Cretaceous	this contribution
**Afrotropical Realm**			
*Ephemerellina* Lestage, 1924	1^E^	Cape Province of RSA	[17, 22, 35–39]
*Lestagella* Demoulin, 1970	1	Cape Province of RSA	[17, 22, 35, 38–41]
*Lithogloea* Barnard, 1932	2	Cape Province of RSA	[17, 22, 35, 37, 39]
*Manohyphella* Allen, 1973	1^F^	Madagascar	[17, 30, 42, 43]
*Nadinetella* McCafferty & Wang, 1998	2	Cape Province of RSA	[17, 22, 35, 44, 45]

Remarks: A – the specimen was attributed to the genus *Teloganella* in its original description [29]; B – as subgenus of the genus *Teloganodes* in [27]; C – series of undescribed taxa associated with *Teloganodes* were recorded from Sri Lanka, Thailand, Malaysia, and South India [46]; D – for details of proposed species and generic synonymy see [24] and [28]; E – recorded from the Democratic Republic of Congo and RSA is a series of undescribed taxa associated with *Ephemerellina,* which belong to the genus *Lithogloea* [46]; F – for descriptions of larvae and adults along with species synonymy see [42]

Using COI/16S gene combinations of African and Madagascar species of Teloganodidae, the Oriental species *Dudgeodes ulmeri* Sartori, 2008, the Afrotropical genus *Ephemerythus* Gillies, 1960, and the South American monotypic genus *Melanemerella brasiliana* Ulmer, 1920 (covering the families Tricorythidae Lestage, 1942 and Melanemerellidae Demoulin, 1955, respectively), Pereira-da- Conceicoa [17] provided a time-calibrated tree along with paleo distributional events during the Gondwana breakup. These reconstructions support the Gondwanan origin of Teloganodidae.

Recent studies mainly focused on amber inclusions from the Mesozoic and Cenozoic and demonstrated the importance of vicariance, dispersal, and extinction in shaping the distribution of several mayfly families, which are linked to the breakup of Pangea and East Gondwana (e.g., Ameletopsidae Edmunds, 1957, Baetiscidae Edmunds & Traver, 1954, and Vietnamellidae Allen, 1984) [14, 47–49]. They notably proposed, based on ancestral range estimates, geological, and palaeontological evidence, that biotic exchanges between fragments of East Gondwana and Asia occurred via “biotic ferries” such as the West Burma Terrane (WBT) and the Indian Plate [18, 50]. This hypothesis aligns with the “out-of-India” hypothesis, which proposes that biotic dispersal occurred via the Indian Plate during the Cretaceous and Paleogene [51, 52]. This scenario, with a particular emphasis on the role of the WBT, has been discussed in the context of freshwater faunal elements of Gondwanan affinity discovered within the Burmese amber biota [53–57]. However, the extent to which these events and hypotheses can be generalised across mayfly lineages, and whether they can be used to explain the faunal composition of the mid-Cretaceous Burmese amber biota, a most significant mid-Cretaceous Lagerstätte, remains to be determined.

Over the past decade, intensive studies of mayfly inclusions in Burmese amber have revealed 11 families, 12 genera, and 16 species [58]. Recently, Godunko et al. [48] characterised this fauna as a composite of Laurasian taxa, Pangaean relicts, and lineages with a possible Gondwanan origin (e.g., Vietnamellidae), and proposed a biogeographic scenario linking these latter elements to the breakup of East Gondwana and the drift of WBT. However, the Burmese amber biota alone is insufficient for reconstructing the evolutionary history and historical biogeography of East Palearctic and Oriental lineages. The Indian Plate, as a much larger insular “biotic ferry”, drifted northwards after separating from Gondwana, transporting its biota as an isolated continental fragment across the Tethys [18, 51, 59–62]. Upon colliding with Asia, it is believed to have contributed significantly to the regional fauna. Despite its importance for biogeographic studies, the fossil invertebrate record of the Indian subcontinent remains scarce, with most findings representing terrestrial taxa [63]. This scarcity hampers efforts to quantify the influx of lineages brought by the Indian Plate, although growing documentation of known fossiliferous deposits is beginning to address this gap. Among these, the Eocene Cambay amber (54.5 Ma [64]) stands out as a crucial source of palaeontological data, preserving a diverse assemblage of invertebrates from tropical warm and humid climatic conditions of the Early Eocene Climatic Optimum [65–68]. Although aquatic insects are rare in Cambay amber, adults of Chironomidae Newman, 1834 (Diptera) suggest the presence of locally separated, diverse aquatic and semi-aquatic paleohabitats [65–70].

Recently, the first mayfly from Cambay amber has been described as *Aikahika veta* Sroka et al., 2025 (Atalophleboculata: Leptophlebiidae Banks, 1900), confirming the potential of this amber to preserve such delicate insects [63]. With more than 500 extant species and 100 described genera, this family today features considerable diversity and apparently originated in Gondwana, although before the discovery of *A. veta*, any fossil evidence in the Asian Cenozoic was missing to document the historical biogeography of this group [71–73]. Another fossil mayfly from the Indian subcontinent, *Teloganella gurhaensis* Agnihotri et al., 2020 was described from a compressed single larva from the Palana Formation (Gurha lignite mine, Rajasthan, India) [29]. However, its systematic placement was uncertain; morphological evidence presented herein suggests it belongs to the Pantricorythi Kluge, 2004 (see below).

Agnihotri et al. [29] indicated the period between Late Palaeocene and Early Eocene as the estimated geological age of the Palana Formation, while other authors suggested an age from approximately 66–56 Ma in the Early Palaeocene [74, 75] to 57–54 Ma [76]. In addition to mayfly larva [29], osteoglossid and lepisosteid freshwater fish have been found here [77], as well as spiders (Araneidae Clerck, 1758), cockroaches (Blattodea), a larva of a riffle beetle (Elmidae Curtis, 1830), and benthic water bugs (Aphelocheiridae Fieber, 1851) among other invertebrates [76, 78–81].

Both Cambay amber and Palana Formation sediments were formed between 55 and 65 Ma and 42–55 Ma at a time slightly preceding the collision of the Indian subcontinent with Asia, leading to the uplift of the Himalayas [60]. Under the “Out-of-India” hypothesis (see [61] for more details), Gondwanan taxa could have distributed further north and east in the period after the Indian Plate collision with Asia. If so, among the Cambay amber and Palana Formation fossils, faunal elements closely related to Gondwana should be found. However, according to Sroka et al. [63], there is almost no evidence for the transfer of ancient Gondwana fauna by the Indian Plate, which may be related to their extinction due to extensive volcanic activity on the one hand, and partial isolation of the subcontinent in the Mesozoic and Cenozoic during the northward drift on the other hand [60, 61]. Therefore, the exchange and dispersal of ancient Indian Plate fauna with surrounding areas may have occurred along island chains or land bridges prior to the collision with Asia [63].

Here, we focus on the systematic position and biogeographic significance of new fossil evidence regarding the family Teloganodidae. The larval holotype of *T. gurhaensis* from the Cenozoic of the Indian Subcontinent is re-examined, and a new genus *Bharataganodes* **gen. nov.** is established for this species, which is placed within the family Teloganodidae. In addition, the first fossil adult record of Teloganodidae is described herein as *Chibiphemera cretalota* **gen. & sp. nov.**, based on a single male imago from mid-Cretaceous Burmese amber. Morphological affinities between *Chibiphemera* **gen. nov.** and Afrotropical Teloganodidae are discussed, together with the significance of the WBT and Indian Plate in the northward transfer of Gondwanan biota to Asia.

## Results

### Systematic palaeontology

**Class** Insecta Linnaeus, 1758

**Subclass** Pterygota Lang, 1888

**Order** Ephemeroptera Hyatt & Arms, 1891

**Family** Teloganodidae Allen, 1965

*Chibiphemera* **gen. nov.** = unnamed taxon associated with the family Vietnamellidae (in [48]: p. 30, fig. 9C)

**Fig. 1:**
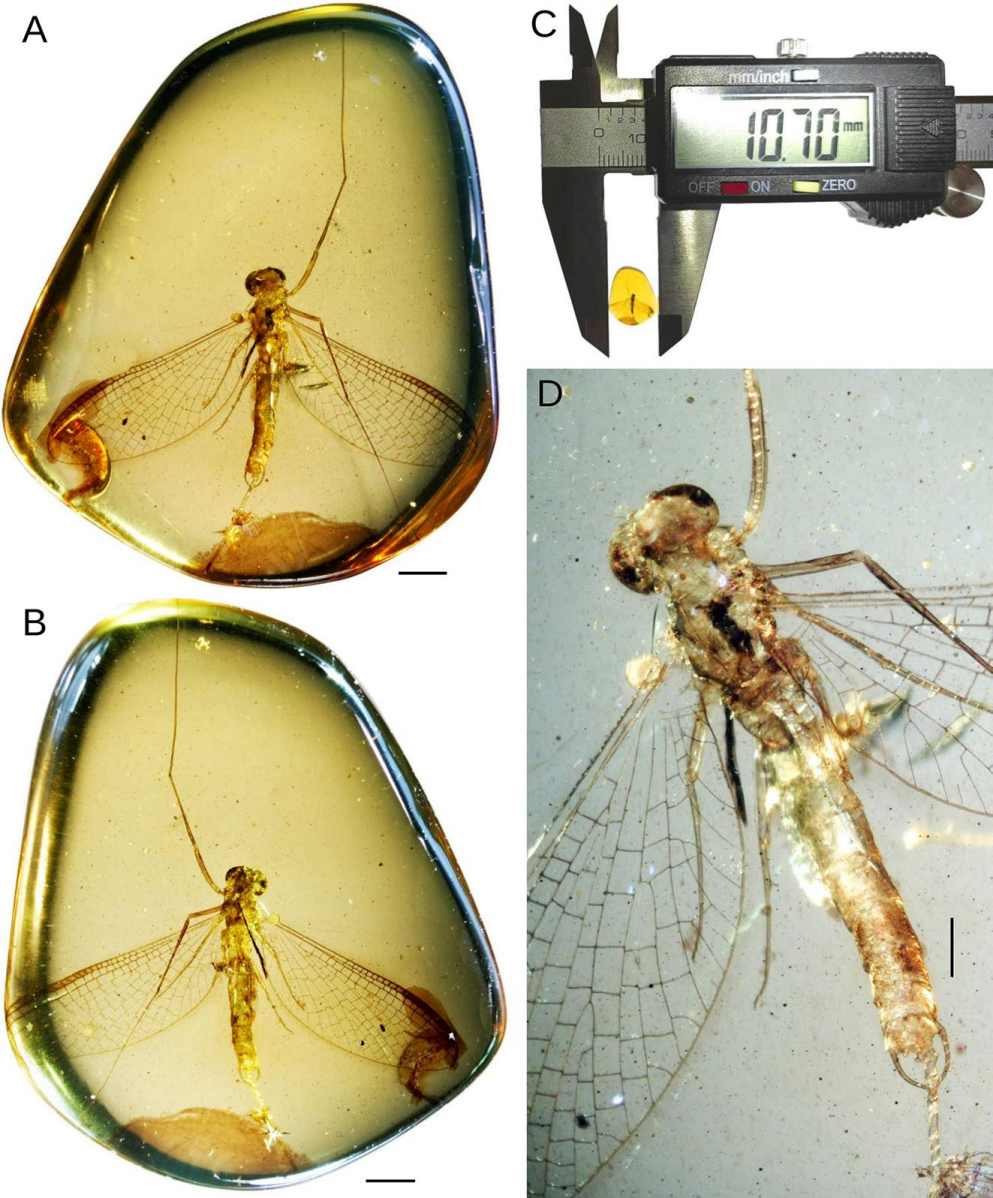
*Chibiphemera cretalota* **gen. & sp. nov.**, holotype, male imago, mid-Cretaceous Burmese amber (Myanmar). **A–C** Entire piece of amber with embedded holotype in (**A**) dorsal and (**B**, **C**) ventral view. **D** Body in dorsal view. Scale bars: (**A**, **B**) 1.0 mm; (**C**) without scale); (**D**) 0.5 mm

LSID urn:lsid:zoobank.org:act:04A0011C-1186-4AE7–B141-768913804A5D

**Derivation of name.** The generic name *Chibiphemera* **gen. nov.** is composed from two parts, the Chinese term 赤壁 [*Chìbì*, meaning “Red Cliff”] and *phemera* as the part of the Latinised Greek *ephemera* [ἐφήμερος, “short-lived”], which is a frequently used suffix for the genera of the order Ephemeroptera. *Chìbì* belongs to a historically and culturally important place on the Yangtze River, which is famously commemorated in the prose 赤壁賦 [*Chìbì Fù*, “Red Cliff Rhapsody”] by the poet Su Shi of the Song dynasty. In this classic example of Chinese literature with a history dating back 900 years, the fleeting, changeable and rapidly passing nature of the winged stages of mayflies is reflected.

**Type species.** *Chibiphemera cretalota* **sp. nov.**

**Species composition.** Monospecific.

**Diagnosis.** *Male imago*.

*Measurements* (**i**) Body length 4.95 mm; forewings 5.64–5.80 mm; hind wings 0.47–0.49 mm, as long as 0.08 of forewings.

*Head* (**ii**) Upper portion of compound eyes large, widely rounded, contiguous medially; division of compound eyes into two portions poorly distinguishable.

*Thorax* (**iii**) Mesonotum short; MNs distinct, transversal centrally, slightly bent forward distally; LPs moderately curved laterally, not reaching MPs, touching PSp near its anterolateral edge; FSp not contiguous, well separated posteriorly.

*Forewings* (**iv**) not narrow, anterior margin jagged; cross venation rich, well developed; free small intercalaries distributed between iMP–A2 only; 16–17 simple cross veins in pterostigmatic area; RS forked basally, approx. at 0.14 of its length; 16–21 cross veins between Sc and RA; MP forked after 0.40–0.42 of its length; iMP free, short; cubital sector with one unforked and one secondary forked vein arising from CuA; CuP arises from wing base, smoothly curved distally; 2–3 cross veins between A1 and CuP; A1 smoothly curved distally; A2 arises from A1.

*Hind wing* (**v**) with straight anterior margin; costal process prominent, sharply pointed, situated strongly proximally; cross venation not developed; RSp arises from MA, MA and MP not forked.

*Legs* (**vi**) Both foreleg pretarsal claws pad-like, blunt apically; both middle and hind legs pretarsal claws hooked apically.

*Abdomen* (**vii**) Gill socket vestiges recognizable on segments II–V; paracercus well developed.

*Genitalia* (**viii**) Large median projection of styliger plate widely rounded apically; pedestals elongated; three distal segments of forceps strongly elongated and slender; segment II longest, 2.7x as long as segment III, and 5.9x as long as oval-shaped segment IV; penis lobes widely separated by V-shaped cleft, stick-like basally, bent inwards apically.

*Chibiphemera cretalota* **sp. nov.**

LSID urn:lsid:zoobank.org:pub:090A9F26–A0C0-4D5F-BAE5-5E8722068DF2

**Fig. 2:**
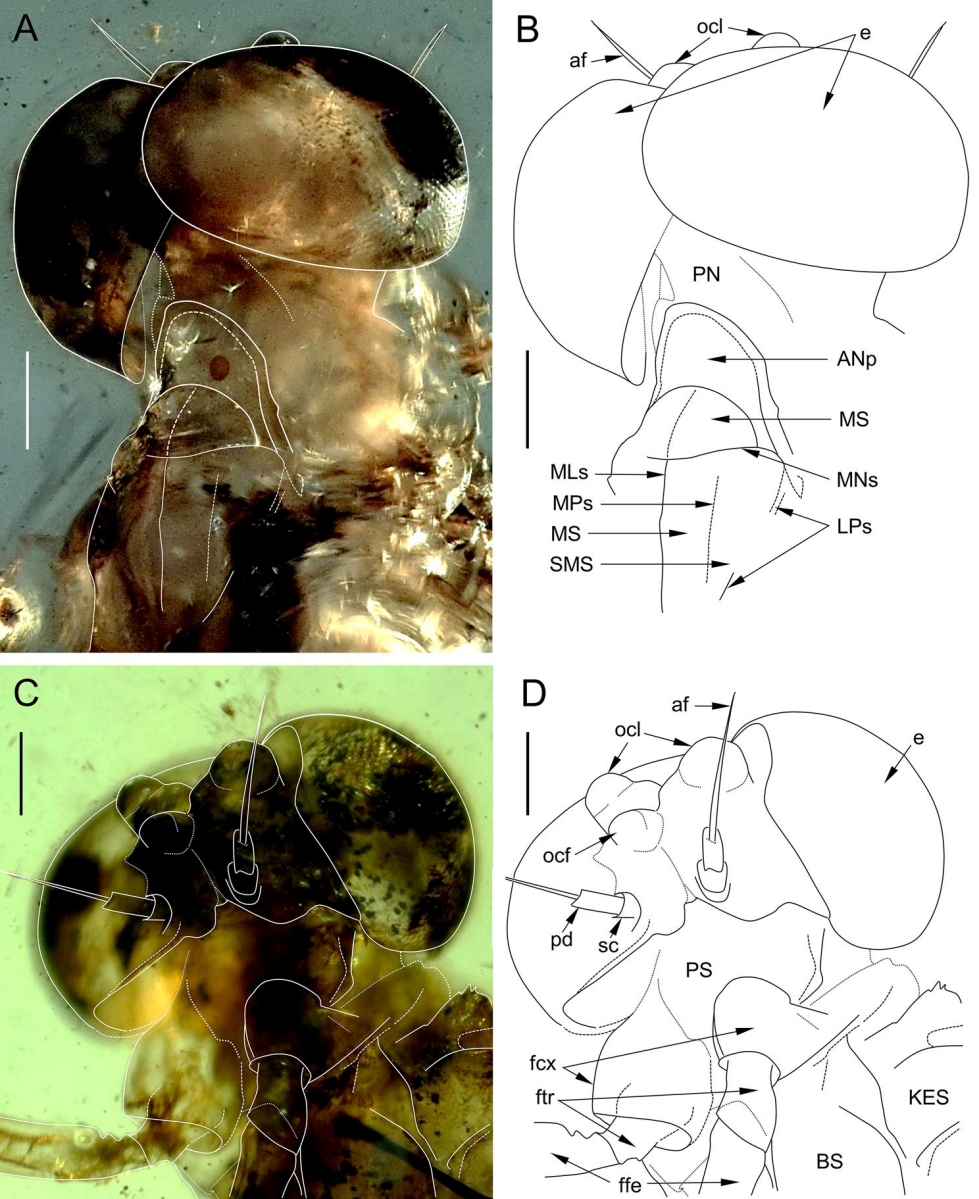
*Chibiphemera cretalota* **gen. & sp. nov.**, holotype, male imago, mid-Cretaceous Burmese amber (Myanmar). **A**, Head and anterior part of thorax in dorsal view from right side; some details of the body structure are marked with white solid and contour lines (scale bar 0.1 mm); **B**, Same as in Fig. 2A, line drawing (scale bar 0.1 mm); **C**, Head and anterior part of thorax in ventral view from left side; some details of the body structure are marked with white solid and contour lines (scale bar 0.1 mm); **D**, Same as in Fig. 2C, line drawing (scale bar 0.1 mm). Abbreviations. *Head*: *af* – antennal flagellum; *e* – eyes; *ocf* – frontal ocellus; *ocl* – lateral ocelli; *pd* – pedicle; *sp* – scape. *Thorax*: *ANp* – anteronotal protuberance; *BS* – basisternum of mesothorax; *KES* – katepisternum; *LPs* – lateroparapsidal suture; *MLs* – median longitudinal suture; *MNs* – mesonotal suture; *MPs* – medioparapsidal suture; *MS* – medioscutum; *pn* – pronotum; *PS* – prosternum; *SMS* – submedioscutum. *Legs*: *fcx* – forecoxa; *ffe* – forefemur; *ftr* – foretrochanter

**Fig. 3:**
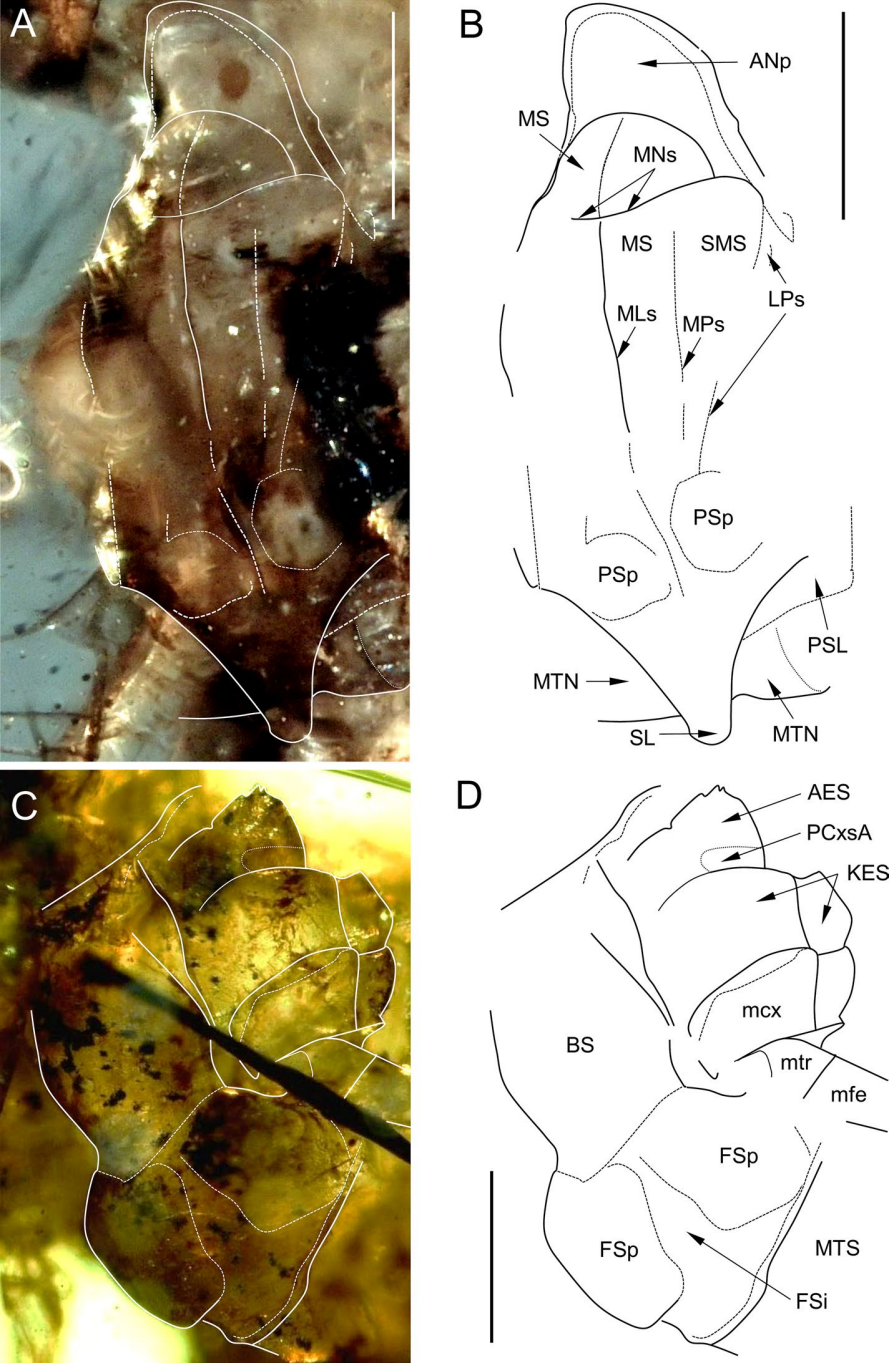
*Chibiphemera cretalota* **gen. & sp. nov.**, holotype, male imago, mid-Cretaceous Burmese amber (Myanmar). **A**, Mesonotum in dorsal view; some details of the mesonotum structure are marked with white solid and contour lines (scale bar 50 µm); **B**, Same as in Fig. 3A, line drawing (scale bar 50 µm); **C**, Mesosternum in ventral view; some details of the mesosternum structure are marked with white solid and contour lines (scale bar 0.1 mm); **D**, Same as in Fig. 3C, line drawing (scale bar 0.1 mm). Abbreviations. *Mesonotum*: *ANp* – anteronotal protuberance; *BS* – basisternum of mesothorax; *KES* – katepisternum; *LPs* – lateroparapsidal suture; *MLs* – median longitudinal suture; *MNs* – mesonotal suture; *MPs* – medioparapsidal suture; *MS* – medioscutum; *PSL* – parascutellum; *SL* – scutellum. *Mesosternum*: *AES* – anepisternum; *BS* – basisternum; *FSi* – furcasternal impression; *FSp* – furcasternal protuberance; *KES* – katepisternum. *mtn* – metanotum; *MTS* – metasternum. *Legs*: *fcx* – forecoxa; *ffe* – forefemur; *ftr* – foretrochanter; *mcx* – middle coxa; *mtr* – middle trochanter; *mfe* – middle femur

**Fig. 4:**
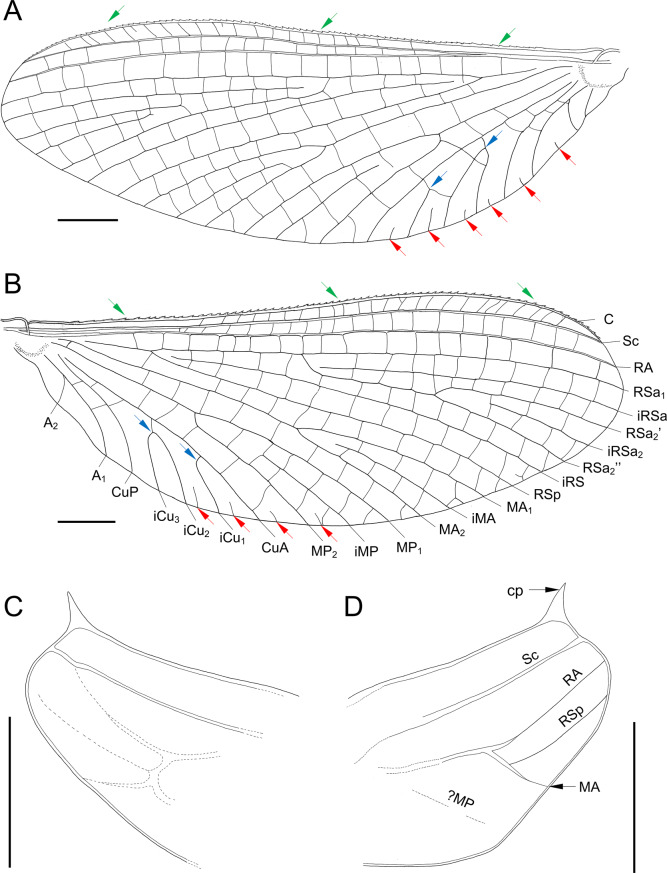
*Chibiphemera cretalota* **gen. & sp. nov.**, holotype, male imago, mid-Cretaceous Burmese amber (Myanmar). **A**, Left forewing in dorsal view; jagged edge marked by green arrows; cubital intercalaries [iCu] are marked by blue arrows; free small intercalaries are marked by red arrows (scale bar 0.5 mm); **B**, Right forewing in dorsal view; markings are same as in Fig. 4A (scale bar 0.5 mm); **C**, Left Hind wing (scale bar 0.25 mm); **D**, Right Hind wing (scale bar 0.25 mm). Abbreviations. *Hind wing*: *cp* – costal process

**Fig. 5:**
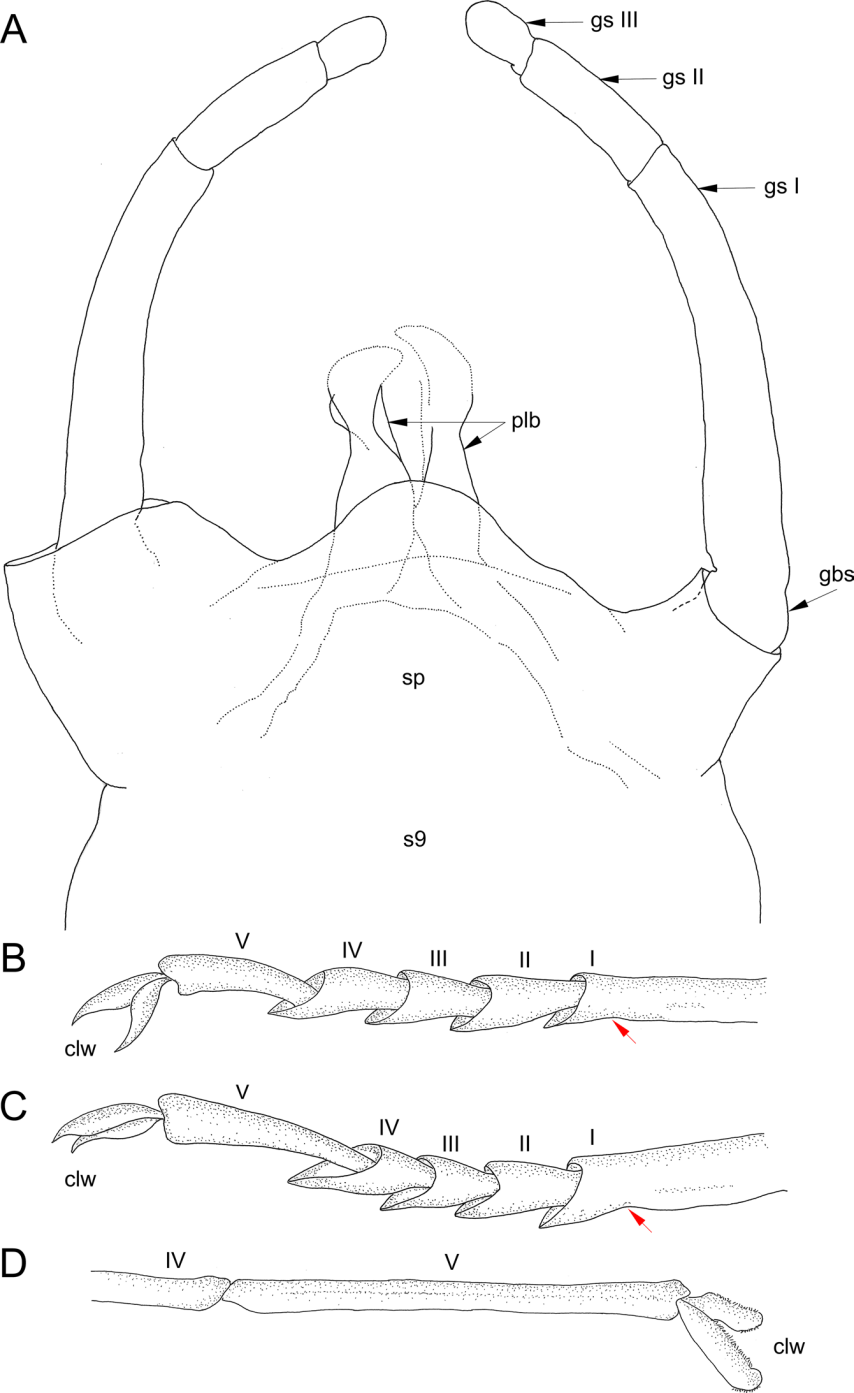
*Chibiphemera cretalota* **gen. & sp. nov.**, holotype, male imago, mid-Cretaceous Burmese amber (Myanmar). **A**, Genitalia in ventral view (without scale); **B**, Left middle tarsus; fusion of the shortened tarsal segment I with tibia marked by red arrow (without scale); **C**, Left Hind tarsus; markings same as in Fig. 5B (without scale); **D**, Right foretarsus (without scale). Abbreviations. *Genitalia*: gbs – basal segment of gonostylus; gs I – gonostylus segment I; gs ii – gonostylus segment ii; gs iii – gonostylus segment iii; plb – penis lobes; *s9* – sternum 9; *sp* – styliger plate; *Legs*: *I–V* – tarsomeres I–V; *clw* – pretarsal claws

Figures 1, 2, 3, 4, 5; Tables 2 and 3

**Table 2:** Measurements of the holotype of *Chibiphemera cretalota* **gen. & sp. nov.** (male imago; ZDL coll., catalogue number T25L07001, Tainan city, Taiwan, China)

Characters	mm	Characters	mm
Length of body	4.95	Length of tibia	1.14
Length of right foreleg	5.82*	Length of tarsus	0.28
Length of femur	1.30	Segment I	0.04
Length of tibia	1.92	Segment II	0.06
Length of tarsus	2.60*	Segment III	0.06
Segment I	0.62	Segment IV	0.05
Segment II	0.76	Segment V	0.07
Segment III	0.82	Length of right hind leg	0.92*
Segment IV	0.40	Length of femur	0.92
Segment V	–	Length of tibia	–
Length of left foreleg	6.43	Length of tarsus	–
Length of femur	1.08	Segment I	–
Length of tibia	1.96	Segment II	–
Length of tarsus	3.39	Segment III	–
Segment I	0.66	Segment IV	–
Segment II	0.76	Segment V	–
Segment III	0.78	Length of left hind leg	2.22
Segment IV	0.85	Length of femur	0.86
Segment V	0.34	Length of tibia	0.98
Length of right middle leg	2.29	Length of tarsus	0.38
Length of femur	0.84	Segment I	0.07
Length of tibia	1.18	Segment II	0.08
Length of tarsus	0.27	Segment III	0.07
Segment I	0.04	Segment IV	0.06
Segment II	0.06	Segment V	0.10
Segment III	0.05	Length of right forewing	5.64
Segment IV	0.05	Length of left forewing	5.80
Segment V	0.07	Length of right hind wing	0.47
Length of left middle leg	2.34	Length of left hind wing	0.49
Length of femur	0.92	Hind/Fore wings length ratio	0.08

*– preserved part

**Table 3:** Summary of characters of extant and extinct adults of Teloganodidae, with focus on †*Chibiphemera* **gen. nov.** and the representatives of the *Ephemerellina* group of genera^A^ distributed in South Africa and Madagascar (including *Ephemerellina* Lestage, 1924, *Lithogloea* Barnard, 1932, *Lestagella* Demoulin,1970, *Nadinetella* McCafferty & Wang, 1998, *Manohyphella* Allen, 1973)

Characters	†*Chibiphemera *gen. nov.	*Ephemerellina* Lestage, 1924^B^	Lestagella Demoulin,1970^C^	Lithogloea Barnard, 1932^D^	Manohyphella Allen, 1973^E^	Nadinetella McCafferty & Wang, 1997^F^
	Extinct; mid-Cretaceous Burmese amber, Upper Albian, max. age is 98.79 ± 0.62 Ma	Extant; Afrotropical Region (South Africa and Madagascar)				
Adult [sex]	male imago	males | females	males | females	males | females	males | females	males | females
*Measurements*						
Body length [mm]	4.95	7.50–8.50 | 8.00–9.50	4.90–5.20 | 5.36–5.50	6.00–6.50 | 6.50–7.00	6.50–7.10 | [5.00–6.00]^G^ 6.10–6.20	6.00–7.30 | 7.00–8.50
Forewings length [mm]	5.64–5.80	9.20–10.20 | 10.50–11.80	5.35–5.50 | 5.80–6.40	6.25–7.00 | 6.50–7.40	6.80–8.10 | [10]^H^	6.00–7.00 | 6.50–8.00
					8.70–9.80	
Hind wings length [mm]	0.47–0.49	1.46–1.64 | 1.68–1.89	1.23–1.28 | 1.15–1.30	0.90–1.10 | 0.95–1.15	1.11–1.15 |	0.85 | 0.94
					0.97–1.02	
Hind/Forewings length ratio	0.08	0.14–0.18	0.23 | 0.21	0.15	0.15 | 0.11	0.12–0.14
Forewings [width/length ratio]	0.35–0.38	0.34–0.38	0.34–0.36	0.32–0.34	0.30–0.32 |	0.34 | 0.34–0.36
					0.32–0.35	
Hind wings [width/length ratioж costal process not included]	0.60–0.63	0.60–0.70	0.56–0.58	0.62–0.66	0.60–0.62 |	0.65–0.67
					0.64–0.66	
*Head*						
Compound eyes of male [shape]	large	large	large	large	large	large
Compound eyes of male [division into upper and lower portions]^I^	present, poorly distinguishable	present	present	present	present	present
Upper portion of male compound eyes [structure]	contiguous	nearly contiguous	nearly contiguous	nearly contiguous	nearly contiguous/contiguous	contiguous
*Thorax*						
Mesonotum [shape]	relatively short	relatively elongated	relatively elongated	relatively elongated	relatively short	elongated
Mesonotal suture [MNs; shape]	distinct, transversal centrally, slightly bent forward distally	distinct, transversal	distinct, transversal	distinct, transversal	distinct, transversal	distinct, transversal
Lateroparapsidal suture [LPs; shape of posterior end]	curved laterally	curved laterally	curved laterally	curved laterally	curved laterally	curved laterally
Furcasternal protuberances [FSp; shape]	not contiguous	not contiguous	not contiguous	not contiguous	not contiguous	not contiguous
Furcasternal protuberances [FSp; inner margins]	tapered anteriorly	tapered anteriorly	tapered anteriorly	tapered anteriorly	tapered anteriorly	tapered anteriorly
*Forewing*						
Forewing [shape]	not narrow	relatively narrow	relatively narrow	relatively narrow	relatively narrow	relatively narrow
Jagged edge [location]^J^	anterior margin	absent	posterior margin	posterior margin	absent	absent
Free small intercalaries [presence]	present	present	present	present	present	present
Free small intercalaries [location]	iMP–A_2_	Sc–CuP	RSa–CuP	Sc–CuA	RA–CuA	RA–CuP
Cross venation	rich, well developed	moderately developed	moderately developed	moderately developed	moderately developed	moderately developed
Pterostigma [number of cross veins]	16–17	12–16	8–10	14–18	8–10	8–9
Pterostigma [shape of veins]	simple	simple and forked	simple and forked	simple and forked	simple and forked	simple and forked
Sc–RA [number of cross veins]	16–21	10–16	4–5	15–19	3–10	4–8
R sector [number of free intercalaries]	absent	4–6	5–6	6–8	3–6	6–7
RS furcation [respectively to vein length]	0.14	0.2	0.28	0.2	0.18–0.20	0.22–0.25
MA fork [shape]	nearly symmetrical	slightly asymmetrical	slightly asymmetrical	slightly asymmetrical	nearly symmetrical	slightly asymmetrical
MA fork [place of furcation]	0.55–0.57	0.5	0.5	0.5	0.42–0.47	0.58–0.60
MP fork [shape]	slightly asymmetrical	slightly asymmetrical	slightly asymmetrical	slightly asymmetrical	nearly symmetrical or slightly asymmetrical	slightly asymmetrical
MP fork [place of furcation]	0.40–0.42	0.24	0.3	0.21	0.35–0.37	0.20–0.22
MP_2_[shape]	long, connected to MP_1_	long, connected to MP_1_	long, connected to MP_1_	long, connected to MP_1_	long, connected to iMP or MP_2_	shor, connected to iMP
iMP [length respective to MP_2_]	shorter	shorter	shorter	shorter	shorter or longer	longer
iMP [proximal end]	free	free	free	free	free or attached to MP_2_	attached to MP_1_
Cubital sector [number of secondary bifurcate veins arising from CuA]	1	absent	absent	absent	absent	absent
Cubital sector [number of unforked veins arising from CuA]	1	1	0–1	1	0–1	absent
Cubital sector [number of free small intercalaries]	2–4	0–1	2–3	0–1	0–1	0–1
Cubital sector [number of free long intercalaries]	1	1	2–3	2–3	2–3	up to 4
CuP [shape distally]	smoothly curved	moderately curved	moderately curved	sharply curved	sharply curved	sharply curved
CuP [shape basally]	arises from wing base	arises from wing base	arises from CuA	arises from wing base	arises from wing base	arises from wing base
CuP–A_1_[number of cross veins]	2–3	1	1	1	1	1
CuP–A_2_[number of free small intercalaries]	0–2	absent	absent	absent	absent	1–2
A_1_[shape distally]	smoothly curved	moderately curved	moderately curved	moderately curved	moderately curved	curved
A_2_[location]	arises from A_1_	arises from A_1_	arises from A_1_	arises from A_1_	arises from A_1_	arises from wing base
Long free intercalaries [location]	Cu sector (1)	R sector (0); MA sector (0–1); MP (0–1); Cu sector	R sector (0–2); MA sector (0–1); MP (1–2); Cu sector	R sector (0); MA sector (0–1); MP (0–1); Cu sector	R sector [1–3]; MA sector [1, 2]; MP [1, 2]; Cu sector [2, 3]	R sector [1, 2]; MA sector [1, 2]; MP [2]; Cu sector [up to 4]
		(2–3)	(2–3)	(2–3)		
*Hind wing*						
Wings [shape]	relatively narrow, with straight anterior margin	oval, with convex anterior margin	oval, with convex anterior margin	oval, with convex anterior margin	oval, with convex anterior margin	oval, with slightly convex anterior margin
Jagged edge [location]^J^	absent	absent	posterior margin	posterior margin	absent	absent
Costal process [shape]	prominent, sharply pointed	not protruding, triangular-shaped	not protruding, triangular-shaped	not protruding, triangular-shaped	prominent, sharply pointed	not protruding, widely rounded
Costal process [location]	app. 1/4 from the tip of wing	app. 2/3 from the tip of wing	app. 1/2 from the tip of wing	app. 2/3 from the tip of wing	app. 1/3 from the tip of wing	app. 3/5 from the tip of wing
Vein triads [number]	absent	2	2	2	absent	2
RSp [location basally]	arises from MA	free	free	free	arises from MA or free	arises from MA
MA fork	absent	absent	absent	absent	absent	absent
MP fork	absent	present	present	present	absent	present
Cubital venation	? absent	present	present	present	present or absent	present
*Legs*						
Forelegs: pretarsal claws [shape]	similar, both pad-like, blunt apically	dissimilar	similar, both pad-like, blunt apically	dissimilar	dissimilar	similar, both pad-like, blunt apically
Middle and hind legs: pretarsal claws [shape]	similar, both hooked apically	dissimilar	dissimilar	dissimilar	dissimilar	dissimilar
*Abdomen*						
Gill socket vestiges [location on terga]	II–V	II–VI	II–IV	II–IV	II–V	II–V
Tubercle vestiges [location on terga]	absent	absent	absent	absent	absent	III–V
Paracercus	developed	developed	developed	developed	developed	developed
*Genitalia [male imago]*						
Styliger plate [shape of median projection]	large, strongly convex, widely rounded	not prominent, almost flat	moderately convex, rounded	not prominent, almost flat	moderately convex, rounded	moderately convex, rounded or triangular shaped
Gonostylus [number of distal segments]	3	3	3	3	3	3
Gonostylus [length ratio of distal segments I–III]	1.00/0.37/0.17	1.00/1.32/0.30	1.00/1.76/0.58	1.00/1.22/0.37	1.00/1.56/0.50	1.00/1.42/0.34
Gonostylus distal segment III [shape]	oval	ovoid	ovoid	ovoid	ovoid [cone-shaped]	nearly oval
Penes lobes [shape]	deeply separated, stick-like, bent inward apically	straight, moderately slender, almost fused except at apex	straight, almost fused except at apex	trapezoidal, almost fused except at apex	straight, almost fused except at apex	straight medially, almost fused except at apex, lobes expanded laterally

*Remarks*: A – The *Ephemerellina* group of genera reflects the plesiomorphon Ephemerellina/g1 sensu Kluge [82], see also [83]. The genus *Indoganodes* Selvakumar, Sivaramakrishnan & Jacobus, 2014 described from South India and Sri Lanka [25, 26] is not included in this analysis

B – based on material of SMNS and IE BC CAS, and [36, 37, 84]

C – based on material of SMNS and IE BC CAS, and [22] (pp. 392, 403–405, 433, fig. 86), [40] (pp. 452–461, figs. 2–5), [17] (pp. 31–36 figs. 3.2.A, B, 3.3.–3.5.), [35]

D – based on material of SMNS and IE BC CAS, and [37] (pp. 252–253, fig. 42a–d), [22] (pp. 392, 401–403, 433, figs. 85, 91), [85] (pp. 264, 271), [35]

E – based on material of SMNS and IE BC CAS, and [42] (pp. 4–8, figs. 1–5), [86] (pp. 42–46), [62, 43], (pp. 160–163, figs. 2, 3), [22, 87&^89^]

F – based on material of SMNS and IE BC CAS, and [38] (pp. 633–635, fig. 8b–d); as *Ephemerellina barnardi* Lestage, 1924 [*partim*], tested by [45] (p. 125), [44] (pp. 12–15, figs. 8–10), [22] (pp. 392, 399–401, 433, figs. 84, 90), [35]

G – female imago body length according to [43]

H – female imago forewing length according to [43]

I – dioptic compound eyes according to [86]

J – wing character was depicted by [40] (fig. 3b)

**Material examined.** *Holotype.* Male imago, mid-Cretaceous Burmese amber, housed in the collection of Zhendong Lian (Tainan City, Taiwan, China) under inventory number T25L07001.

**Derivation of name.** The specific epithet *cretalota* is a constructed feminine adjective combining *creta* [Latin for “chalk”, referring to the Cretaceous geological period as a time of Burmese amber origin] and *lota*, a feminised form inspired by lotus [Latinised from Greek lōtos]. We used the lotus as a cross-cultural symbol of purity, rebirth, and transience in ancient literature and art. In ancient Sanskrit, the lotus represents the sacred sunrise and sunset, spiritual transformation, and renewal. The same is mentioned in relation to the nature of adult mayflies in many historical and artistic sources. This species name also holds personal significance for one of the authors, since lotus is a homonym of Zhendong Lian’s surname. The name *cretalota* is considered to be a feminine adjective, matching the grammatical gender of the genus *Chibiphemera* **gen. nov.** in accordance with Article 31.2 of the International Code of Zoological Nomenclature (ICZN).

**Diagnosis.** *Male imago*. As for *Chibiphemera* **gen. nov.**, as monospecific.

**Generalities.** Relatively well-preserved and almost complete imaginal specimen, in pale, translucent amber, embedded in dorsoventral aspect; both forelegs are complete; right and left middle legs, and right hind leg are incomplete, with some tarsomeres missing; paracercus preserved, cerci missing (Fig. 1; Table 2).

**Description.** *Male imago* (Figs. 1, 2, 3, 4, 5; Tables 2 and 3). *Colours*. Preserved colour of specimen yellowish-brown to brown, with inconspicuous brownish-black to black maculae on eyes, head, and thorax. Thorax ventrally slightly paler than dorsally, its lateral margins covered by blackish maculae. Wings pale, hyaline, translucent, yellow to light brown; forewing pterostigma frosted. Legs yellow to dark brown, darker than body; tarsi slightly paler than tibiae and femora. Abdominal segments yellowish-brown to intensively brown, genitalia of same colour; paracercus yellow-brown, blackish distally.

*Measurements*. Body length 4.95 mm [*as preserved*]; forewing length 5.64–5.80 mm; hind wing length 0.47–0.49 mm. Maximum forewing width 0.35–0.38x of maximum length; hind wing 0.08x of forewing length. For other measurements and comparative data see Tables 2 and 3.

*Head*. Facial keel small, not protruding anteriorly, dirty brown to black. Antennae yellow to dirty brown, with small black dots; flagellum slightly paler than scape and pedicle. Ocelli covered by inconspicuous maculae; frontal ocellus smaller than lateral ocelli. Division of compound eyes into two poorly distinguishable portions, better recognisable on left eye; upper portion of compound eyes well developed, large and widely rounded, contiguous medially; lower portion of eye narrow, height less than 0.12 × of upper portion; facets of compound eyes hexagonal (Figs. 1C and 2).

*Thorax*. Thoracic terga darker than sterna, yellowish-brown to dark brown, with inconspicuous black maculae; pleurae yellow to light brown. No traces of specific pigmented areas on mesonotum. Thoracic sterna paler than terga, light brown. Mesonotum relatively short; mesoscutellum not elongated; mesonotal suture [MNs] distinct, transversal centrally, slightly bent forward distally; points of MNs crossing with medioparapsidal sutures [MPs] not visible; MPs slightly convergent towards posterior scutal protuberance [PSp]. Lateroparapsidal suture [LPs] elongated, moderately curved laterally, not reaching MPs, touching PSp near its anterolateral margin. Anterior paracoxal suture of mesothorax [PCxsA] short, not reaching sternum; anepisternum [AES] and katepisternum [KES] well separated, reaching sternum. Basisternum of mesonotum [BS] relatively short; furcasternal protuberances [FSp] not contiguous, separated by a median furcasternal impression [FSi], which is widened posteriorly (Figs. 2 and 3).

*Wings*. Forewings mostly hyaline, translucent, frosted by dirty brown colour in pterostigmatic area only. Cross veins slightly paler than longitudinal veins; cross venation well developed, yellow or yellowish-brown in basal half of forewing to distinctly brown in distal half, darkest between C and RA (Fig. 1A, C).

*Forewings* not narrow, anterior margin jagged. Pterostigmatic area with 16–17 simple cross veins. Cubital brace well preserved, strongly arched. C, Sc, and RA well visible throughout their length, brown to dark brown. RS forked near base, approximately at 0.14 of its length; 16–21 cross veins between Sc and RA; no free small intercalaries in R sector. MA nearly symmetrical, forked at 0.55–0.57 of its length; numerous cross veins in MA sector; MP slightly asymmetrical, forked after 0.40–0.42 of its length; MP_2_ long, connected to MP_1_, iMP free, shorter than MP_1_ and MP_2_, and connected to it by 3–4 cross veins; free small intercalaries distributed iMP and A_2_ only. Tornus close to CuA, weakly pronounced. Cubital sector with one unforked and one secondarily forked vein arising from CuA; three intercalary veins of different length running from CuA towards posterior margin of wing; no cross veins between main stout cubital intercalaries, and 2–4 free intercalaries between CuA and CuP (at least one free intercalary vein approx. twice as long as others); CuA and CuP closely approximated and connected near wing base; CuA arises from wing base, nearly straight; CuP arises from wing base, smoothly curved distally; 2–3 cross veins between CuP and A_1_; up to two free small intercalaries between CuP and A_2_; A_1_ arises from wing base, smoothly curved distally; A_2_ arises from A_1_ (Fig. 1A, C and 4A, B; Tables 2 and 3).

*Hind wing* hyaline, translucent, relatively narrow, with straight anterior margin, as long as 0.08 of forewing length; anterior and posterior wing margins not jagged. Costal process prominent, sharply pointed, situated close to wing apex. Vein triads and cross venation not developed; RSp arises from MA, MA and MP not forked; cubital venation not distinguishable (Fig. 4C, D; Tables 2 and 3).

*Legs* well preserved except for right hind leg. Forelegs paler than middle and hind legs, yellow to dirty brown. Artificial dark brown to blackish maculae covering surface of legs as a result of fossilization. Tibiopatellar suture present on basal 1/3 length of middle and hind legs, absent on forelegs. First tarsomere of middle and hind legs shortest, fused with tibia (for measurements of leg segments see Table 2). Pretarsal claws of forelegs both pad-like, blunt apically; all pretarsal claws of middle and hind legs apically hooked (Fig. 5B–D; Tables 2 and 3).

*Abdominal segments* completely preserved; vestiges of gill socket on segments II–V; no vestiges of tubercles on surface of terga. Paracercus well developed.

Styliger plate with large, median, widely rounded apically projection, markedly protruding above anterior margin, nearly as long as pedestals of gonostyli; both pedestals elongated, slightly tapered apically. Gonostyli with three distal segments; first segment longest, moderately bent inwards distally; lateral margins of segment II nearly parallel; segment III oval; length ratio of distal gonostyli segments I–III: 1.00/0.37/0.17. Penis lobes stick-like basally, bent inwards apically, widely separated by V-shaped cleft (Figs. 1A and 5A; Tables 2 and 3).

*Bharataganodes* **gen. nov.**

LSID: urn:lsid:zoobank.org:act:5E8108B8-6193-4B89–AB3B-99C30D9E6F0B

**Derivation of name.** The generic name *Bharataganodes* **gen. nov.** is derived from the Sanskrit word *Bharata* (भरत), an ancient and poetic name for India, referring to the country of origin of the type material. The suffix -*ganodes* is used in some extant Oriental genera of the family Teloganodidae. The generic name is masculine in gender.

**Type species.** *Bharataganodes gurhaensis* (Agnihotri, Chandra, Shukla, Singh & Mehrotra, 2020) **comb. nov.**

**Species composition.** Monospecific.

**Diagnosis.** *Larva*.

*Measurements* [as preserved] (**i**) Body length 12.37 mm; thorax/abdomen length ratio is 0.68; maximal length of cerci 6.13 mm.

*Head* (**ii**) prognathous, widely rounded anteriorly; a row of long stout setae along anterior and lateral margins; a dense row of setae in clypeal region.

*Thorax* (**iii**) with broad pronotum, length about 0.45x of its maximal width; anterolateral angle of pronotum protruded and pointed apically.

*Legs* (**iv**) Forefemora slightly asymmetrical; inner and outer margins clearly convex, each nearly symmetrical; no anteroapical projection or hump on outer margin; foretibia moderately widened distally, tapered in proximal part; foretarsi longer than foretibiae, with robust and hooked pretarsal claws. Middle and hind femora asymmetrical, inner margin slightly convex or nearly straight; outer margin clearly convex.

*Abdomen* (**v**) Prominent posterolateral projections visible on terga VI–IX, largest projections on terga VIII–IX; no traces of median tubercles on abdominal terga.

Gill I small, styliform, moderately narrowed distally, attached to segment I close to its outer margin medially, and directed posteriorly-medially.

Three caudal filaments; paracercus as long as cerci.

*Bharataganodes gurhaensis* (Agnihotri, Chandra, Shukla, Singh & Mehrotra, 2020) **comb. nov.**

= *Teloganella gurhaensis* Agnihotri et al., 2020 (in [29]: p. 138, fig. 2, 3)

LSID urn: lsid:zoobank.org:pub:F5CFE110–D935-43F6–A18D-49F381E8E8D1

**Fig. 6:**
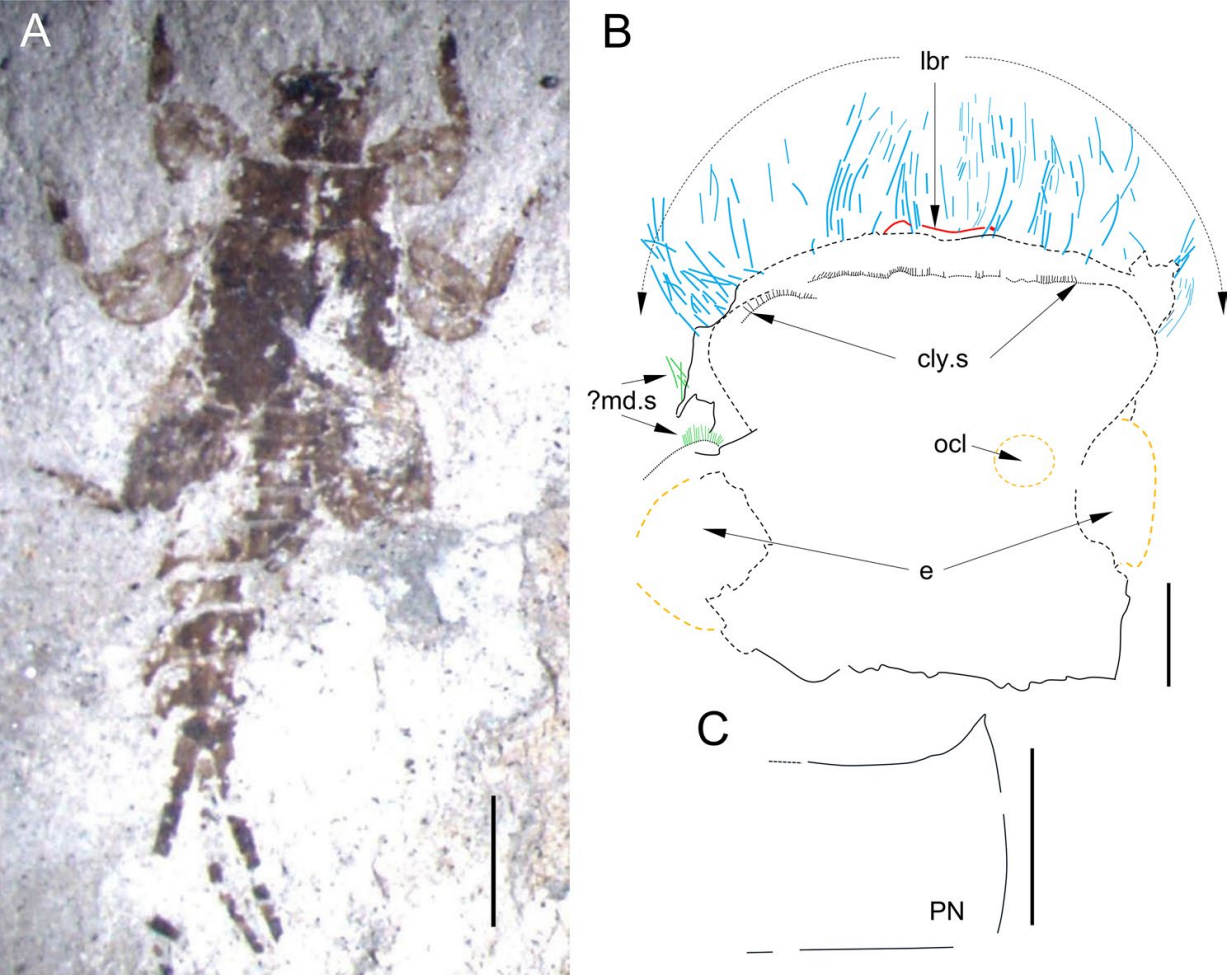
*Bharataganodes gurhaensis* **comb. nov.**, holotype, larva, late Palaeocene–early Eocene, Palana Formation (India). **A**, General view of body in dorsal view (scale bar 2.5 mm); **B**, Head in dorsal view; details of setation marked by coloured lines, namely long setae along anterior margin in blue, putative clypeal setae in black and putative mandibular setation in green; anterior margin of labrum marked in red (scale bar 0.2 mm); **C**, Right side of pronotum in dorsal view (scale bar 0.5 mm). Abbreviations. Head: *cly.S* – clypeal setation; *e* – eyes; *lbr* – labrum; *?md.S* – putative mandibular setation; *ocl* – ocellus [right]. Thorax: *pn* – pronotum

**Fig. 7:**
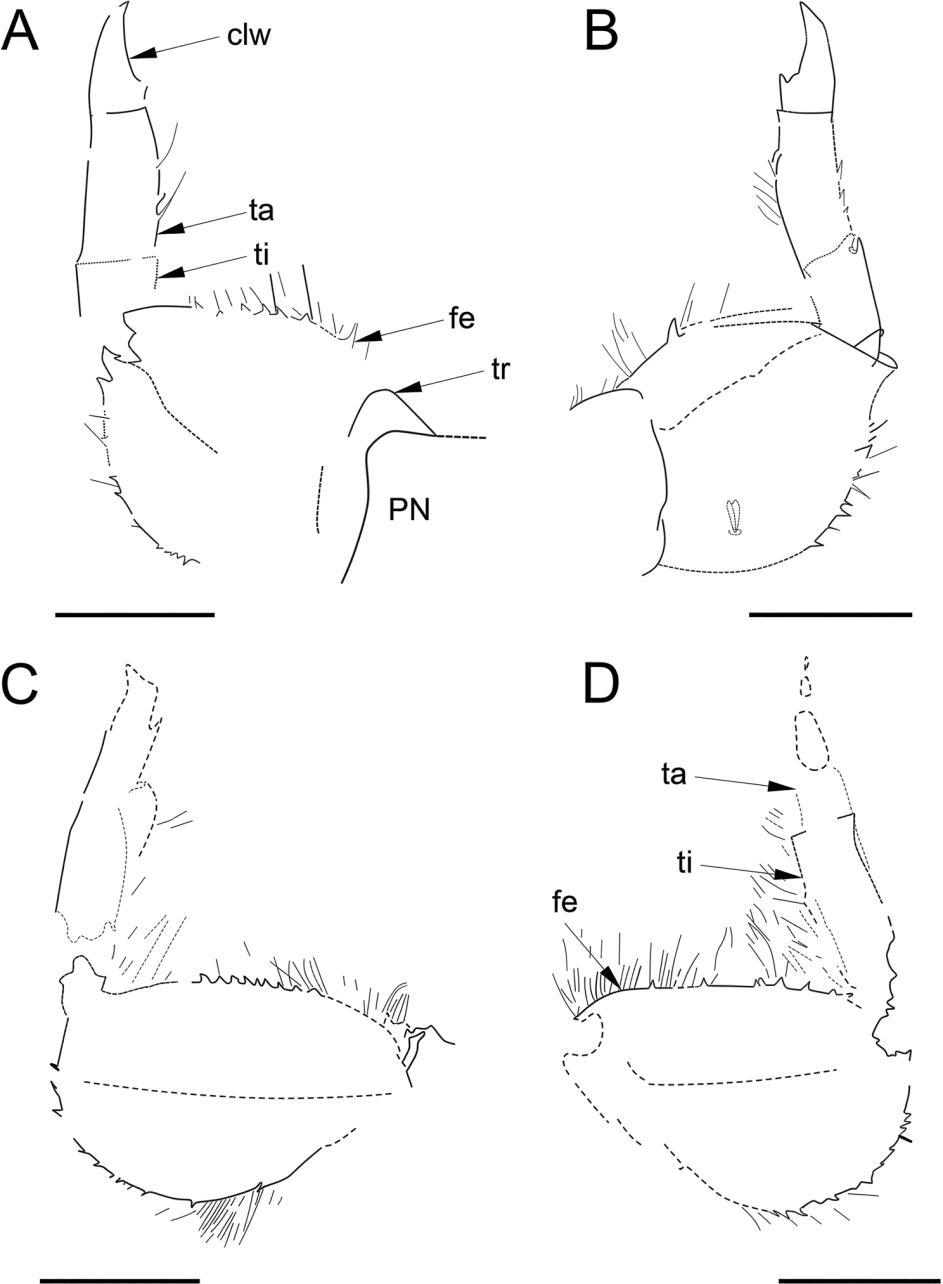
*Bharataganodes gurhaensis* **comb. nov.**, holotype, larva, late Palaeocene–early Eocene, Palana Formation (India). **A**, Left foreleg in dorsal view (scale bar 0.5 mm); **B**, Right foreleg in dorsal view (scale bar 0.5 mm); **C**, Left middle leg in dorsal view (scale bar 0.5 mm); **D**, Right middle leg (scale bar 0.5 mm). Abbreviations. *Legs*: *clw* – pretarsal claws; *fe* – femur; *ta* – tarsus; *ti* – tibia; *tr* – trochanter. *Thorax*: pn – pronotum

Figures 6–7, Table 4

**Table 4:** The summary of larval characters of extant and extinct Teloganodidae and Teloganellidae. Only the morphological characters preserved in the fossil larva of *Bharataganodes gurhaensis* **comb. nov.** are discussed and compared here with those of extant representatives (except for the distribution and structure of gills in extant genera)

Characters	Teloganodidae												Teloganellidae
	†*Bharataganodes* gen. nov.	EPH^J^	LES^E^		LIT^H^		NAD^I^		MAN^D^		TELO^A^, DUD^A^, DER^A^	IND^C^	TELLA^B^
	Extinct; late Paleocene-early Eocene, max. age is 57 Ma	Extant; Afrotropical Region (South Africa and Madagascar)									Extant; Oriental Region (China, India, Indonesia, Malaysia, Philippines, Sri Lanka)		Extant; Oriental Region (India, Malaysia)
Larvae [sex]	? male	m | f		m | f		m | f		m | f		m | f	m | f	m | f	m | f
*Measurements*													
Body length [mm]	12.37*	10–12.0		5.0–6.5		7.0–9.0		6.8–8.2		5.0–6.0	TELO: and DUD: 4.0–7.0; DER: 3.0–5.0	8.5–12.5	4.0–5.0
Cerci length [mm]	6,13*	12.0–15.0		3.0–4.5		4.0–6.0		4.5–6.5		3.0–4.0	TELO: and DUD: 2.5–8.0; DER: 3.0–5.5	12.0–14.0	4.0–4.5
Thorax/abdomen length ratio	0.68	0.75–0.85		0.65–0.75		0.70–0.80		0.70–0.80		0.75–0.80	0.60–0.85	0.62–0.66	0.50
*Head*													
Eyes [shape and structure]	relat. large, widely separated	moder. large, separated		moder. large, separated		moder. large, separated		moder. large, separated		relat. large, separated	TELO: moder large; DUD: large; DER: moder. large; all well separated	moder. large, widely separated	not large, widely separated
Anterior margin [shape]^F^	irregularly rectangular, widely rounded	widely rounded		widely rounded		widely rounded		widely rounded		widely rounded	widely rounded to dome-shaped	widely rounded	widely rounded, dome-shaped
Anterior margin [setation]	dense, thin and stout, long	very sparse, thin and short		dense, thin and stout, markedly long		sparse, thin and stout, short		sparse, thin and stout, short		moder. dense, thin, relat. long	TELO: dense, thin and stout, long; DUD: variable in density and length, stout and hair-like; DER: very dense and long, thin and stout	moder. dense and short	dense, thin and stout, long
Lateral margins [setation]	dense, thin and stout, moder. long	mainly absent		dense, stout and thin, short		mainly absent		mainly absent		sparse, very short	TELO: as above; DUD: as above; DER as above	mainly absent	moder. develop., short
*Mouthparts*													
Labrum [shape of incision of anterior margin]	no deep incision	widely, deeply incised		widely incised		shallow incision		deeply incised		deeply incised	widely incised	deeply incised	relat. deeply incised, broadly emarginate
Outer margin of mandibles [setation]	dense, thin and stout, long	a few, hair-like, short		sparse, hair-like, long		sparse, hair-like, not long		sparse, hair-like, not long		sparse, hair-like, long	a single robust seta (sometimes additional smaller bristle)	dense, short and medium	relat. dense, long
*Thorax*													
Pronotum [anterolateral margin]	protruded, pointed apically	not protruded		not protruded		not protruded		not protruded		not protruded	not protruded or slightly protruded	not protruded	slightly protruded, widely triangular
*Legs*													
Forefemur [shape]	greatly expanded, widest centrally, slightly asym.	relat. narrow, asym.		moder. expanded, strongly asym.		relat. narrow, asym.		relat. narrow, slightly asym.		greatly expanded, widest prox., strongly asym.	TELO: moder. to greatly expanded, flattened, strongly to moder. asym; DUD: the same; DER: the same, strongly asym.	moder. expanded	greatly expanded distally, strongly asym.
Forefemur [marginal setation]	dense denticulation, long, thin and stout setae**	sparse, thin and stout, very short along i.m.; dense, much longer prox. on o.m.		moder. long, stout, one submarg. row going to o.m. prox.; scales, spat. setae and short hairs along i.m.		sparse, thin and stout, not elong., along i.m.; dense, moder. long along o.m.		sparse, thin and stout, very short along i.m.; dense, markedly longer prox. on o.m.		thin and stout, moder. long, domin. i.m.	rows of stout setae of diff. length and shapes, small scales and hair-like setae on o.m.; hair-like setae of diff. length on i.m.	stout and short	stout, mainly long
Forefemur [marginal teeth and scales]	present	present		present		present		present		present	TELO, DUD present; DER absent	present	absent
Foretibia [shape]	widest distally	slightly widened distally		nearly parallel margins		nearly parallel margins		nearly parallel margins		slightly widened distally	slightly widened or nearly parallel	widest distally	widest prox.
Foretibia [marginal setation]	dense, long, thin and stout setae; small spines on o.m.**	thin, not elong., domin. o.m.		sparse, short hair-like		thin and stout, not elong., domin. o.m.		thin and stout, not elong., domin. o.m.		thin and stout, moder. long, domin. i.m.	thin and stout setae of diff. length, dense on o.m.; small scales and hair-like setae on i.m.	sparse hair-like setae	dense, thin and stout, long
Foretarsus [shape]	nearly parallel margins	nearly parallel margins		nearly parallel margins		nearly parallel margins		nearly parallel margins		nearly parallel margins	nearly parallel margins	moder. widened distally	moder. widened distally
Foretarsus [marginal setation]	sparse, stout, moder. long, i.m.	similar to foretibia		sparse, very short hair-like		sparse, thin and stout, relat. short, domin. o.m.		similar to foretibia		sparse, thin and stout, short and long	sparse, thin and stout, short and long	stout, small scales and setae, domin. i.m.	thin and stout, long, domin. o.m.
Foretarsus [to foretibia length]	longer	shorter		shorter		shorter		shorter		shorter	shorter	shorter	shorter
Foreleg pretarsal claws [shape]	moder. hooked, robust, promin. hump prox.; no preserved teeth	moder. elong. and hooked, no hump prox.; row of 5–7 teeth		elong., no distinct hump prox.; row of 4–6 teeth, dist. tooth largest; row of 4 small subapical setae		moder. elong and hooked; row of 4–6 teeth; row of up to 5 small subapical setae		elong., moder hooked; more than 20 teeth in two rows; row of up to 5 small subapical setae		moder. elong., no distinct hump prox.; row of sparse, blunt teeth	TELO, DUD: hooked, relat. stout, 3–4 median teeth, prox. hump present or absent; 1–2 subapical teeth; two rows 3–6 thin setae; DER: narrow, hooked, hump prox.; 3–4 median teeth, 2 subapical teeth; two rows of 3–4 thin subapical setae	hooked, no distinct hump prox.; 5–8 teeth	moder. hooked, promin. hump prox.; no teeth
Middle femur [shape/setation]	asym./dense denticulation and long setae**	nearly sym./similar to forefemur		slightly asym./dense, stout and thin, long prox., short distally		nearly sym./similar to forefemur		nearly sym./similar to forefemur		slightly asym./ similar to forefemur	broad, flattened, asym. to slightly asym/similar to forefemur; DER: all groups of setae dense and very long	slightly asym./ similar to forefemur	strongly asym./ stout, mainly long
Middle tibia [shape/marginal setation]	margins parallel or widened distally/dense, long, i.m.	slightly widest distally/ similar to foretibia		parallel margins/sparse, short hair-like		parallel margins/ similar to foretibia		parallel margins/ similar to foretibia		parallel margins/ thin and stout, moder. long, domin. o.m.	slightly widened or nearly parallel/similar to foretibia; DER: all groups of setae dense and very long	widest distally/ similar to foretibia	widest prox./thin, long, domin. o.m.
*Abdomen*													
Gill I [presence/shape]^G^	present/sock-like	absent		present/ finger-like		present/ finger-like		present/ finger-like		present/ finger-like	absent	absent	present/peg-like
Gill I [location on tergite I]	attached on projection, close to outer margin medially	–		as in extinct genus		as in extinct genus		as in extinct genus		as in extinct genus	–	–	attached on projection, close to outer margin posteriorly
Gills [presence on segments]	not preserved	I–VI		I–IV		I–VI		I–V		I–V	TELO: II–VI; DUD: II–V; DER: II–IV	II–VI	I–V
Gills [structure]	not preserved	II largest, semi-operc., II–VI two lobes		II largest operc., II–III two lobes, IV no ventral lobe		II largest, semi-operc., II–V two lobes, VI no ventral lobe		II largest, semi-operc., II–IV two lobes, V no ventral lobe		II largest, operc., II–IV two lobes, V no ventral lobe	TELO: II–VI two lobes, VI no ventral lobe; DUD: II–IV two lobes, V no ventral lobe; DER: II–III two lobes, IV no ventral lobe^K^	II largest, operc., II–VI no ventral lobe	II–IV two lobes, V no ventral lobe
Submedian projections on terga [presence/shape]^L^	absent	I–VIII, elong., pointed on II–VII/unpaired		absent		I–VIII (IX)/unpaired		I–VIII, well develop. on III–VI/ mainly paired		I–VIII, well develop. on III–VIII/unpaired	various combinations: mainly on I–X, except DER: I–IV and X no projections, V–IX poorly developer/well developed (except DER), stout, tuberculate or spine-like, unpaired	absent	III (IV)–VIII/paired
Posterolateral projections of terga [presence/setation]	preserved on VI–IX/ long and stout	(III) IV–IX; short, weakly develop./short hairs and scales		II–IX; well develop. IV–IX/ very long, thin and stout		II–IX; V–IX large, acute/sparse, short and stout		(III) IV–IX; short, weakly develop./short hairs and scales		II–IX; well develop. on V–IX/ moder. long, stout	TELO and DUD: II–V weakly develop, VI–IX well develop.; DER: II–IV almost absent; (V) VI–IX well developed/scales; long to very long, thin and stout setae	I–V almost absent, VI–IX promin./ sparse hair-like	II, IV–IX present, III absent/ long, stout and thin
Paracercus [presence/setation]	well develop./ relat. short setae	well develop./sparse, short spines		well develop./ moder. dense, relat. long setae		well develop./ sparse short spines, dense long setae		well develop./ sparse, short spines; sparse, long setae		well develop./ sparse short spines, shortened stout setae	vestigial, one-segmented	well develop./ dense, short, stout scales and setae	well develop./sparse short spines; relat. dense, elongated setae

*Remarks*: A – based on larval material housed at SMNS, and [37] (pp. 252–253, figs.43d–g, as *Ephemerellina* sp. from Cedarberg); [38] (1940: pp. 634–635, larval size and species distribution; probably two species of Teloganodidae were mixed [45]); [39] (p. 76: summary of previous data; gill description and comparison with *Lithogloea*); [44] (pp. 15–16, figs. 37–45: description and figures of larva); [45] (pp. 123–125, fig. 12: placement within *Ephemerellina*, list of synonymies and records; larval details including mouthparts, legs. and fragmented cercus); [41] (pp. 415, 421: larval key); [22] (pp. 397–398, figs. 4, 13, 22, 31, 70, 79: description and discussion of generic larval and adult characters)

**Fig. 8:**
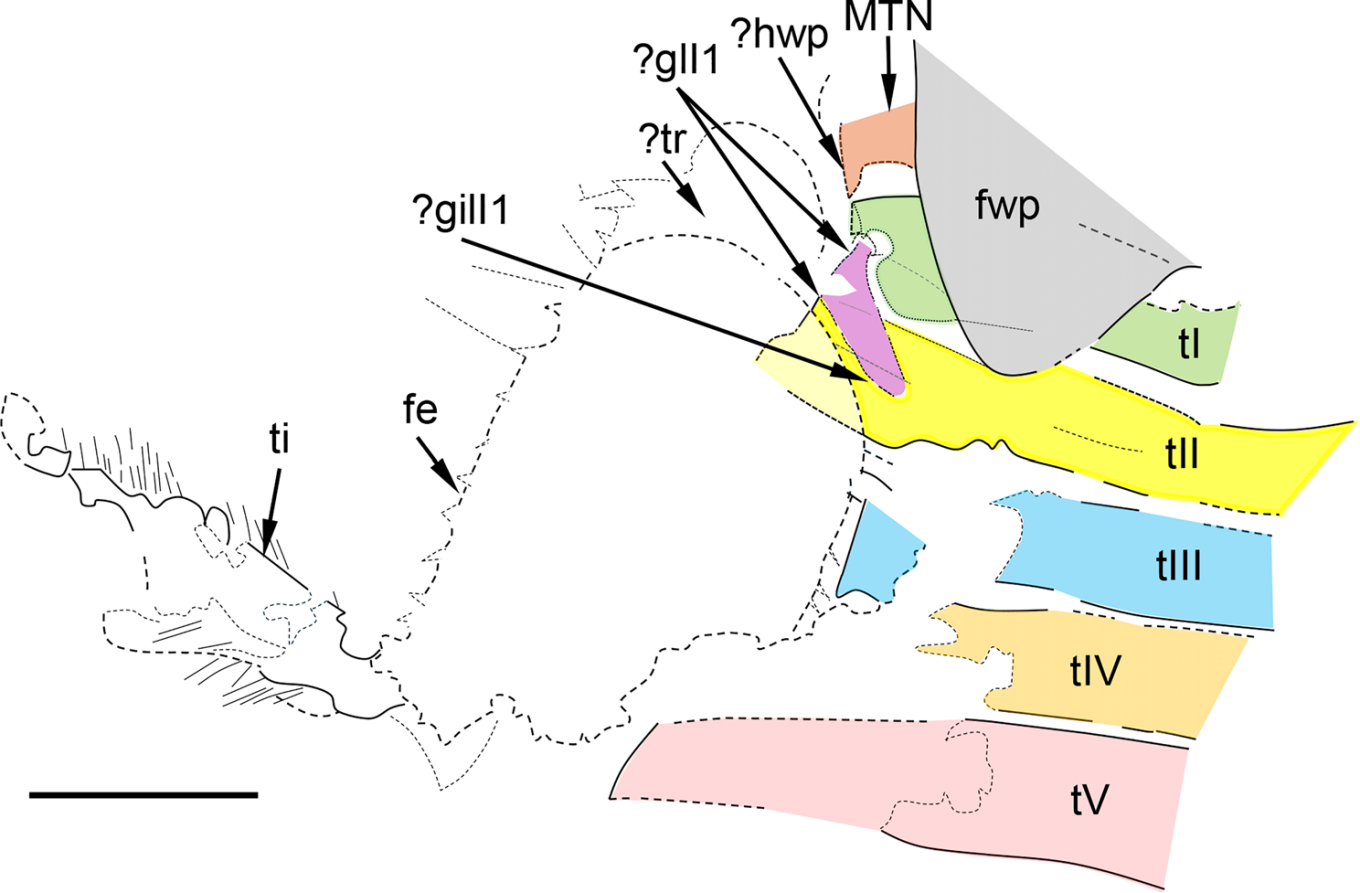
*Bharataganodes gurhaensis* **comb. nov.**, holotype, larva, late Palaeocene–early Eocene, Palana Formation (India). Left side of the body, with preserved left Hind leg, part of thorax and abdominal terga I–V (scale bar 2.0 mm). Abbreviations. *Leg*: *fe* – femur; *ti* – tibia; *?tr* – putative trochanter. *Thorax*: *fwp* – forewing pad; *mtn* – metanotum; *?hwp* – putative Hind wing pad. Abdomen: *?gilI1* – putative gill 1; *tI–tV* – abdominal terga I–V

**Fig. 9:**
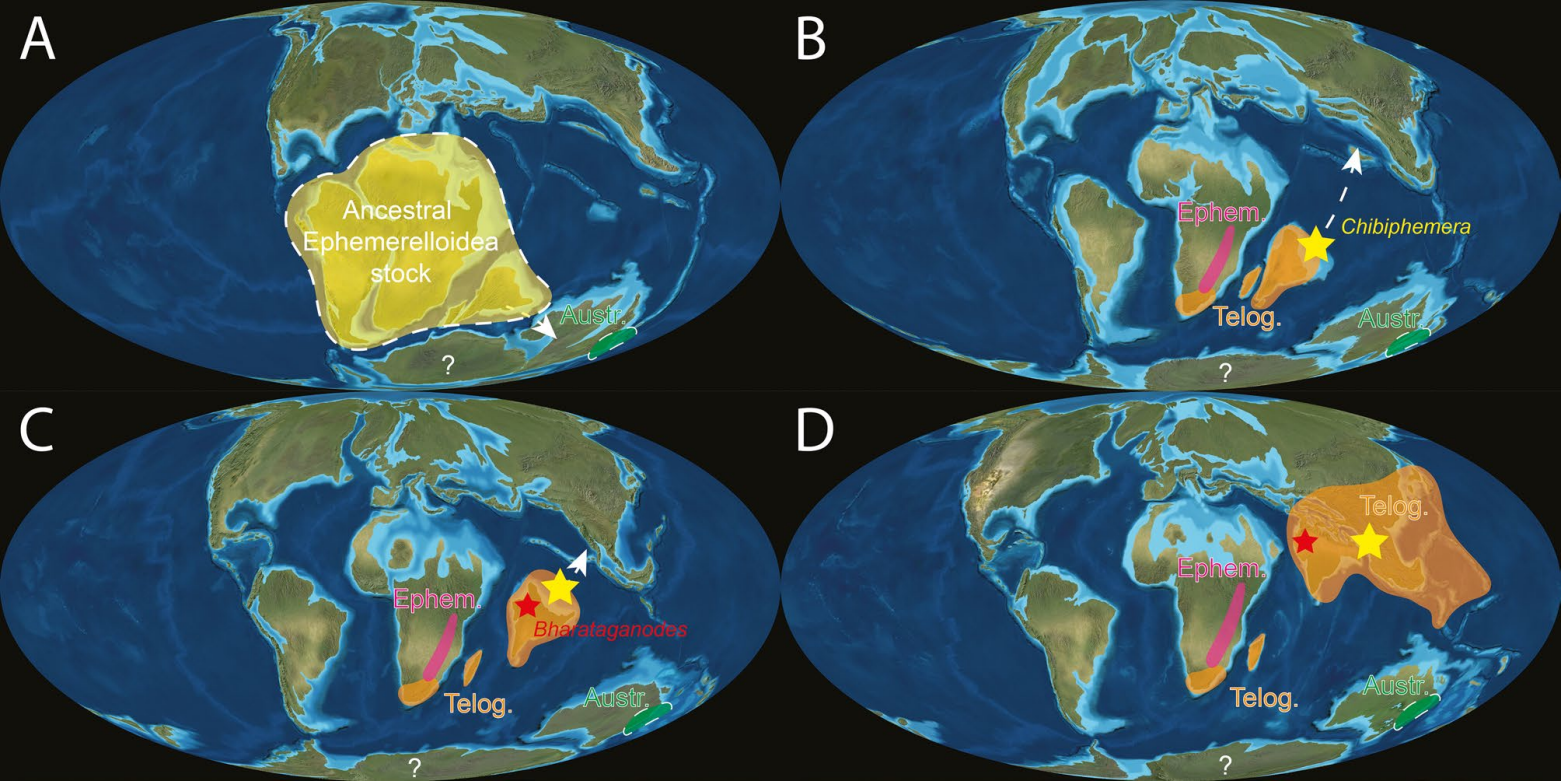
Palaeogeographic scenarios depicting the dispersal and drafting events of Teloganodidae. Interpretation is based on the current distribution of clades and their fossil record. Hypothetical distribution of different Ephemeropteran groups for the: **A**, Early Cretaceous (120 Ma); **B**, Late Cretaceous (∼80 Ma); **C**, Paleocene (∼60 Ma); **D**, Late Eocene (∼40 Ma). Abbreviations. *Austr*. – Austremerellidae; *Ephem*. – Ephemerythidae; *Telog*. – Teloganodidae. White dotted arrows represent potential dispersals. The yellow star represents the Burmese Terrane and *Chibiphemera cretalota* **gen. & sp. nov.** The red star represents the Gurha lignite mine and *Bharataganodes gurhaensis* **comb. nov.** The question mark represents the lack of information for Antarctica. The underlying maps are modified from the Global Palaeogeography and Tectonics in Deep Time series from Colorado Plateau Geosystems Inc. © 2016

B – based on larval material housed at SMNS, and [37] (pp. 253–255, figs. 43–44: initially described and confused with *Lithogloea harrisoni*); [38] (p. 637, fig. 9: described as *Lithogloea penicillata*); [39] (pp. 77–78: brief note on *Lestagella penicillata* originally reported as *Lithogloea,* including information on body size, the presence of long setation on the anterior head margin and gill I, referred to as “small rudimentary processes on the first abdominal segment”; also noted were the flattened forefemur and the absence of long setation on cerci in *L. penicillata*); [45] (pp. 122, 130–132, fig. 15: establishment of the monotypic genus *Lestagella,* listing of larval characters in the key and illustrations of specimens of different ages); [41] (pp. 420–421, pl. VII: same data and illustrations as in [45]); [22] (pp. 392, 403–405, figs. 7, 34, 52, 68: generic larval and adult characters); [40] (pp. 452–460, figs. 2, 6–16: designation of male imaginal lectotype, redescription of larva and adults)

C – based on larval material housed at SMNS, and [90] (pp. 252–255, figs. 43, 44: confused with *Lestagella penicillata*); [38] (p. 636: differences between *Lithogloea harrisoni* and the newly described “*Lithogloea penicillata*”); [39] (pp. 76–77 [92] unnumbered fig.: differences between several Teloganodidae species); [45] (pp. 128–129, fig. 14: as subgenus within *Ephemerellina*) [22]; (pp. 392, 401–403, figs. 6, 15, 24, 33, 72: generic larval and adult characters)

D – based on larval material housed at SMNS, and [44] (pp. 12–15, figs. 2–8: attributed to *Ephemerellina*); [45] (pp. 126, 128: referred both *Nadinetella brincki* and *N. crassi* to *Ephemerellina*, albeit suggesting that the latter may warrant subgeneric status due to the presence of the first pair of gills, which distinguishes it from *E. barnardi*; description of a “Forma *simplex* f. nov.” within *Ephemerellina*, which undoubtedly belongs to the genus *Nadinetella*, as suggested by [22] based on the presence of a double row of claw teeth, lamellate gills on abdominal segments II–V, and variable submedian projections on the abdominal terga, features similar to those found in *N. crassi*); [22] (pp. 392, 399–401, figs. 5, 14, 23, 32, 71, 74–76: generic larval and adult characters)

E – based on [42, 86, 87]

F – based on material mostly housed at NMNH NASU, and [22, 24, 25, 27, 34, 46, 93]

G – based on material housed at NMNH NASU and [25, 26, 34]

H – based on [25, 30–32], including larval characters of the monotypic genus *Janohyphella indica* Selvakumar, Sivaramakrishnan and Jacobus, 2014, recently transferred to *Teloganella*

I – the setation along the anterior margin of the head is often described as a fringe of setae in Teloganodidae (see [22, 24, 40]), and Teloganellidae ([25], under *Janohyphella*).

J – gill I in *Lestagella* was described as “filamentous, three-segmented, with long, thin, fine setae” [40]: fig. 15a; initially a “segmented” gill I was reported for *Lestagella* under the name *Lithogloea harrisoni*, with the basal ‘segment’ possibly referring to the projection of gill I, also described for *Bharataganodes* **gen. nov.** (Teloganodidae) and *Teloganella* (Teloganellidae) ([37]: figs. 43a, 44i)

K – for detailed information on the presence or absence of the cleft on the dorsal lobe of gills III–V, see [27, 28]

L – submedian projections are also referred to as abdominal carina, median paired/unpaired protuberances, spines, or tubercles (see [24, 25, 27, 28, 34, 93])

*Abbreviations*: Teloganodidae: EPH, *Ephemerellina* Lestage, 1924; LES, *Lestagella* Demoulin,1970; LIT, *Lithogloea* Barnard, 1932; NAD, *Nadinetella* McCafferty & Wang, 1998; MAN, *Manohyphella* Allen, 1973; TELO, *Teloganodes* Eaton, 1882; DUD, Sartori, 2008; DER, *Derlethina* Sartori, 2008; IND, *Indoganodes* Selvakumar et al., 2014; Teloganellidae: TELLA, *Teloganella* Ulmer, 1939; asym., asymmetrical; dist., distal; diff., different; develop., developed; domin., dominate on; elong., elongated; i.m., [inner margin]; f, females; m, males; moder., moderately; o.m., outer margin; operc., operculate; promin., prominent; prox., proximal, proximally; relat., relatively; spat., spatulate; submarg., submarginal; sym., symmetrical. * – As preserved. ** – The denticulation along the femora and tibiae of the fore and middle legs in *Bharataganodes* **gen. nov.** may represent remnants of the bases of long stout setae, rather than short sharp spines or scales as observed in other Teloganodidae. Thus, we use this character with caution

**Material examined.** *Holotype.* Larva, late Palaeocene–early Eocene, Palana Formation, Gurha lignite mine of Bikaner (Rajasthan, NW India); BSIP specimen no. 41,813, see also [29].

**Revised diagnosis.** *Larva*. As for *Bharataganodes* **gen. nov.**, as monospecific.

**Generalities.** Relatively well-preserved, almost complete larva, visible in dorsal aspect, collected from light yellow to medium grey clay layers of the Gurha lignite mine in Bikaner district of Rajasthan [29]; body flattened dorsoventrally, partly crushed as a result of compression during fossilisation. Fore- and middle legs are mostly preserved; right hind leg partly damaged, distal part of femur, tibia and tarsus are missing. Three caudal filaments preserved, partly damaged (Figs. 6 and 7).

**Redescription** (modified from [29]). *Larva* (Figs. 6 and 7). *Colours.* Remnants of the putative original cuticular pigmentation are preserved as a dark spot on the anterior part of the head between the eyes; two dark spots of similar size near the posterior margin of the head may represent remnants of the dorsal portion of putatively male larval compound eyes. Symmetrical, elongated, light strip along both sides of the central pronotal suture; a broad, nearly symmetrical, dark V-shaped macula centrally on the pronotum, possibly outlining the remnants of a V-shaped impression; symmetrical light spots also visible anteriorly on the mesonotum, contrasting markedly with the darker wing pads. Dark, elongated triangular maculae visible through the wing pads, especially on the left side of the mesonotum, represent the developing foreprotoptera (forewing anlagen sensu Kluge [46]). The foreprotoptera are fused to the mesonotum at their base and partly along the basitornal margin; supposed imprint of left foreprotoptera with well-defined tornus. Legs paler than thorax, with a dark diffuse central spot on fore- and middle femora; fore- and middle tibiae are darker distally; tarsi of all legs are darkest. Abdominal segments paler than thorax, each preserved segment bearing unspecific dark maculae laterally.

*Measurements*. Body length 12.37 mm (as preserved), head length 1.94 mm; thorax length 4.23 mm; length of abdomen 6.20 mm; thorax/abdomen length ratio is 0.68. Maximal length of cerci 6.13 mm (left cercus, as preserved).

*Head* prognathous, widely rounded and irregularly rectangular anteriorly. Compound eyes visible along lateral margins of head, relatively large; putative remnants of dorsal portion of male compound eyes discernible as two darker spots near posterior margin of head centrally. Traces of ocelli visible on central part of head; contours of relatively large eyes well visible laterally. Anterior and lateral margins of head fringed with a row of long, stout setae; some of these setae possibly related to mouthparts, namely mandibles. Anterior part of head in clypeal region with dense row of setae remnants and their bases, which probably belong to head surface setation. Antennae not preserved; indistinct remnants that may be associated with labrum along anterior margin of head (Fig. 6B).

Other structures of mouthparts either have not been preserved or their remnants cannot be clearly identified.

*Thorax*. Pronotum relatively broad, as long as 0.45x its maximal width, with anterolateral angle protruded and pointed apically (preserved on right side only). Mesonotum well preserved, with wing pads reaching abdominal segment II (left wing pad). Metanotum relatively narrow; no visible projections and tubercles on metanotum surface. Lateral margins of thoracic segments without visible setation (Figs. 6 and 8).

*Legs* well preserved except for right hind leg; trochanteres well recognisable, moderately expanded distally; remnants of minute sparse setae along inner margins of trochanteres. Setation of legs generally poorly preserved except of inner and outer margins; no preserved traces of femoral setae arranged in regular rows, which typical for Pantricorythi as shown by Kluge ([46]: p. 298, fig. 89I).

Femora of all legs robust, clearly widened, with trace of longitudinal ridge. Forefemora 1.10–1.24x as long as wide, middle femur 1.53–1.67x, left hind femur 1.60x [as preserved]; average preserved length ratios of femur, tibia and tarsus [including pretarsal claw; as preserved]: foreleg 1.18/0.50/1.00; middle leg 2.00/1.20/1.00; hind leg ratios not calculated since poorly preserved (Fig. 7).

Forefemora greatly expanded, slightly asymmetrical, widest centrally; inner and outer margins clearly convex; no anteroapical projection or hump on outer margin; both margins covered by strong teeth alternating with setae of different length, longer than teeth; setation of forefemora surface weakly preserved, with trace only one stout setae on surface. Similar shape of setae was depicted for *Indoganodes tschertoprudi* by Martynov & Palatov ([26]: p. 129, fig. 4B, C)]. Foretibia moderately widened distally and tapered in proximal part, markedly shortened; traces of long setae along inner margin; small teeth along outer margin. Foretarsi longer than foretibiae, with nearly parallel margins; pretarsal claws robust, moderately hooked; one prominent hump basally; no traces of preserved row of small teeth along inner margin (Fig. 7A, B).

Middle femora asymmetrical; inner margin slightly convex or nearly straight; outer margin clearly convex; both margins with small, relatively dense denticulation alternating with long setae. Middle tibiae partly preserved, with parallel or slightly widened distally margins; dense setation along inner margin; setation of outer margin not preserved; remnants of putative tibiopatellar suture poorly distinguishable on left middle leg. Tarsi poorly preserved; shape of pretarsal claws indistinguishable (Fig. 7C, D).

Hind legs only partly preserved; left hind femur asymmetrical; inner margin nearly straight; outer margin moderately convex; small, stout denticulation along inner and outer margins; traces of a few long setae on inner margin; Left hind tibia with traces of dense setation along both margins; shape of pretarsal claw indistinguishable (Fig. 8).

*Abdominal* segments partly damaged; terga I–V relatively narrow; robust and prominent posterolateral projections of terga VI–IX visible from left side of abdomen; largest projections on terga VIII–IX; posterolateral projections bearing long, thin setae visible on segments V–IX; no traces of median tubercles on abdominal terga; posterior margin of terga VI and V with unclear traces of minute denticulation; tergite X relatively small.

Putative remnants of gill I small, styliform, moderately narrowed distally, attached to segment I close to its outer margin medially, and directed posteriorly-medially; no preserved remnants of other gill pairs.

Three caudal filaments; paracercus well developed, its preserved part almost as long as cerci; segmentation of caudal filaments distinguishable; remnants of caudal setation present on lateral margins of cerci and paracercus (Figs. 6A and 8).

## Discussion

### Systematic placement

***Chibiphemera*** **gen. nov.**

We assign this extinct genus to the crown group Ephemeroptera based on the markedly reduced hind wings, in combination with the presence of a costal brace in the forewings, which is basally connected to the costal vein. Although the tornus is only weakly pronounced, the forewings of *Chibiphemera* **gen. nov.** exhibit an anteritornous condition, in which the wing tornus is situated between CuA and CuP [46].

*Chibiphemera* **gen. nov.** is assigned to the superfamily Ephemerelloidea. In characterising adult features of Ephemerelloidea (or *Ephemerella*/fg1 sensu Kluge, 2004), Kluge [46] noted the constant proximal connection of CuA and CuP by the cross vein *cua*–*cup*, as well as the consistent connection of CuP and A_1_ by the cross vein *cup*–*aa*, which is situated more proximally than *cua*–*cup*. Additionally, Kluge [46] described CuP as arising from CuA at an acute angle and being bent at the point of connection with *cua*–*cup* (Fig. 4A, B). This forewing venation pattern is shared by all extant and extinct representatives of Ephemerelloidea, including genera of Pantricorythi, whose wings are not highly modified [46, 48, 94, 95].

The venation of the forewing in *Chibiphemera* **gen. nov.** corresponds with that of Ephemerelloidea, as it features CuP arising from CuA, and a series of specific cross veins connecting CuP to both CuA and A_1_. Unlike representatives of Leptophlebioidea McCafferty & Edmunds, 1979, the new genus possesses free, small intercalaries between the longitudinal veins [96]. Furthermore, the mesonotal suture of *Chibiphemera* **gen. nov.** is clearly distinguishable and not transferred backward, as occurs in Caenoidea, Baetoidea, Ephemeroidea and Leptophlebioidea. Lastly, the fossil male imago lacks sockets on the first abdominal segment, a character observed in some other Ephemeroptera. Taken together, these characters support the placement of *Chibiphemera* **gen. nov.** within the superfamily Ephemerelloidea.

Kluge [46] proposed the new circumscriptional name Pantricorythi for a group comprising several families within Ephemerelloidea, namely Vietnamellidae; Austremerellidae McCafferty & Wang, 2000; Melanemerellidae; Teloganodidae; Teloganellidae McCafferty & Wang 2000; Tricorythidae; and Leptohyphidae Edmunds & Traver, 1954. A larval apomorphy of Pantricorythi is the specific arrangement of setae on the dorsal surface of the forefemur (see [46]: p. 316, figs. 89I–J, 100E–F).

Several characters support the placement of *Chibiphemera* **gen. nov.** within Pantricorythi, rather than Ephemerellidae Klapálek, 1909. In Ephemerellidae, the hind wings are well developed, typically measuring 0.2–0.3x the length of the forewings; in contrast, those of Pantricorythi are smaller, usually not exceeding 0.2x the forewing length. In *Chibiphemera* **gen. nov.**, the hind wings are even further reduced, measuring only 0.08x the forewing length (Table 3). Hind wings of Ephemerellidae generally lack a prominent costal process, and two bifurcations (RS and MP) are usually retained. In contrast, Pantricorythi possess a hind wing with a well-developed, often acute, costal process, while its venation is frequently simplified. Vestiges of gill sockets are present from abdominal segment II in *Chibiphemera* **gen. nov.** and other Pantricorythi. In Ephemerellidae, such sockets appear from segment III onwards in the subfamily Ephemerellinae Klapálek, 1909, or from segment IV onwards in the subfamily Timpanoginae Allen, 1984 (see also [46: Table 8] and [94]).

In adults of Pantricorythi, the male gonostyli are typically (though not always) characterised by an elongated first segment; the second segment is the longest, or occasionally equal in length to the first segment. The second segment of gonostyli is directed caudally, or inclined laterally, as observed in *Machadorythus* Demoulin, 1959, *Leptohyphes* Eaton, 1882, *Allenhyphes* Hofmann & Sartori, 1999 and *Tricorythopsis* Traver, 1958. The third segment is the shortest, usually as long as wide, although it may be elongated [46]. However, in contrast to all Ephemerellidae, which always feature gonostyli with strongly shortened first segment, *Chibiphemera* **gen. nov. **is characterised by the first segment of the gonostyli being the longest, more than twice the length of segment II (for comparison with *Eurylophella viscata* (Demoulin, 1968), a fossil representative of the family Ephemerellidae, see [94]: p. 1324, fig. 4C, D, and for *C. cretalota* **sp. nov.** see Fig. 5A).

Currently, Teloganodidae lack a generally accepted circumscription and diagnosis [27]. However, the larvae of Teloganodidae can be clearly distinguished from all other pannote mayflies by shared features of the abdominal gills (for further details, see [30, 46, 97, 98].

McCafferty & Wang [22] proposed a diagnosis for adult Teloganodidae that includes several ancestral features shared with Ephemerellidae, namely the forewing venation pattern, the structure of the mesothorax, and male eyes. They also identified clear differences between Teloganodidae and Ephemerellidae, particularly regarding the relative length of the first segment of the gonostyli, as well as the presence of gill socket vestiges on abdominal segment II (see above). Finally, they noted for Teloganodidae the presence of well-developed free intercalaries along the outer margin of the forewing, and a distinctive cubital venation pattern, bearing one to four main intercalaries.

McCafferty & Wang [22] included the two subfamilies Teloganodinae and Austremerellinae into Teloganodidae. Additionally, they also included *Vietnamella* Tshernova, 1972. Later, they also placed the genus *Manohyphella* in Teloganodidae, while transferring *Vietnamella* and *Austremerella* Allen, 1965 to a newly established family, Austremerellidae [30]. Currently, *Vietnamella* is classified within Vietnamellidae. Besides this extant genus, Vietnamellidae also includes the Cretaceous genus *Burmella* Godunko et al., 2021, described from adults preserved in Burmese amber [48, 95].

*Chibiphemera* **gen. nov.** cannot be assigned to either Vietnamellidae or Austremerellidae. The extinct genus does not exhibit the characteristic hind wing shape, which is strongly rounded in Vietnamellidae. The venation pattern of the hind wings in *Chibiphemera* **gen. nov.** also differs markedly from the single species of Austremerellidae, *Austremerella picta* Riek, 1963. Furthermore, males and females of *A. picta* possess a pair of long, membranous processes (plumidia) on the posterior margin of the mesonotal scutellum [46, 89, 92], which are absent in *C. cretalota* **sp. nov.** In contrast to the fossil genus described here, the adults of both Vietnamellidae and Austremerellidae are also characterised by the presence of gill socket vestiges on abdominal segment VII. In males of *Chibiphemera* **gen. nov.**, such sockets are restricted to segment VI (see [24] and [46]: Table 3).

We attribute *C. cretalota* **sp. nov.** to the family Teloganodidae based on several adult diagnostic characters not shared by other Pantricorythi:

(i) The male compound eyes are large, dioptic, i.e. divided into two portions, with the upper portion contiguous dorsally (in contrast to Ephemerythidae Gilles, 1960, Teloganellidae, Melanemerellidae, most Leptohyphidae, and part of Tricorythidae); this feature can be regarded as a stable diagnostic character of Teloganodidae [31, 32, 46, 99, 100].
(ii) The venation of the forewings is unmodified, with CuP arising from the wing base (in contrast to the taxon Tricoryptera, proposed by Kluge [46] for a group of families including Tricorythidae, Ephemerythidae, Dicercomyzidae Edmunds & Traver, 1954, Machadorythidae Edmunds, Allen & Peters, 1963, and Leptohyphidae, which exhibit modified forewing venation, especially CuP arising near the base of A_1_).
(iii) Free, small marginal intercalaries are well developed in the forewings, with at least one such vein present between each pair of major veins from R to Cu sectors (in contrast to Melanemerellidae and some Ephemerythidae, which bear only 2–3 small intercalaries mainly between R and R–MA, or to Leptohyphidae and Tricorythidae, which have lost marginal intercalaries [46, 85, 101].
(iv) The male gonostyli possess three distal segments (in contrast to some African Pantricorythi, e.g. Dicercomyzidae, Ephemerythidae, Machadorythidae, and Tricorythidae, in which the gonostyli lack the third, distal segment [99, 100, 102, 103]). In the case of Tricorythidae, an identical composition of gonostyli was recently described and illustrated for the first Oriental representative, *Tricorythus meenakshi* Srinivasan et al., 2022, from Tamil Nadu in southern India [104].

Kluge [46] proposed a non-hierarchical taxon Melanemerella/fg1, for the African genera of Teloganodidae, as well as for the Neotropical monospecific genus *Melanemerella brasiliana* Ulmer, 1920. To date, only nymphs and an adult female have been described in *Melanemerella*, whereas the males remain unknown. Interestingly, the Malagasy monospecific genus *Manohyphella keiseri* Allen, 1973 was not included within this taxon. It should also be noted that Melanemerella/fg1 sensu Kluge [46] does not correspond in volume to the subfamily Melanemerellinae Demoulin, 1955 or the family Melanemerellidae, both of which were proposed by other authors exclusively for *M. brasiliana* [85, 98, 105, 106]. More recently, however, Kluge [82] introduced a non-hierarchical name for the plesiomorphon (a group, which is not defined by apomorphic characters) Ephemerellina/g1, as one of the taxa of Pantricorythi [83]. The circumscription of the taxon Ephemerellina/g1 corresponds precisely to the generic composition of Afrotropical Teloganodidae, including the Madagascan species *M. keiseri*, and excluding the Neotropical *Melanemerella*.

Among the adult characters of Ephemerellina/g1, several characters are regarded as plesiomorphic for Ephemerelloidea besides enlarged male eyes: the structure of the mesonotum, including a distinct mesonotal suture, a laterally curved lateroparapsidal suture with characteristic pigmentation of the subimaginal lateral area, and an unmodified scutellum bearing an infrascutellum [82]. Furthermore, vestiges of two bifurcate cubital forewing veins, occasionally reduced to 1–4 intercalary veins, are also listed. Kluge [82] also noted features of the hind wing in Ephemerellina/g1, namely a length ratio of 0.20–0.25 to the forewing length, with Sc reaching the wing apex, a forked RS, and the presence of veins posterior to MP_1_ (the same hind wing characters were listed for Melanemerella/fg1 in [46]).

The morphological characters of *C. cretalota* **sp. nov.** correspond to those listed above for the plesiomorphic group Ephemerellina/g1. Unlike the Oriental Teloganodidae, the lateroparapsidal suture of the mesonotum is clearly curved laterally in both these taxa. Despite its small size, the venation pattern of the hind wings in *C. cretalota* **sp. nov.** more closely resembles that of Afrotropical taxa, particularly in the orientation of Sc (Fig. 4C, D; see also [17, 27, 42]). However, the hind wing venation of the fossil specimen could not be fully described due to the state of preservation. Finally, in contrast to the Oriental Teloganodidae, *Chibiphemera* **gen. nov.**, like other Ephemerellina/g1, possesses a well-developed paracercus (Fig. 1A; Table 3).

Five genera of Teloganodidae are recorded from South Africa and Madagascar. A detailed review of the taxonomic history of Teloganodidae in this region, including their synonymy and distributional patterns, was published by Pereira-da-Conceicao [17]. The relationships between *Chibiphemera* **gen. nov.** and the extant genera *Ephemerellina*, *Lestagella*, *Lithogloea* Barnard, 1932, *Nadinetella*, and *Manohyphella* are discussed in Table 3 in detail, based on morphological characters of both males and females.

*Chibiphemera* **gen. nov.** can be readily distinguished from extant Afrotropical genera of Teloganodidae by the notably smaller size of the male body and wings (Table 3). Additionally, the forewing to hind wing ratio in the fossil genus is 0.08, whereas in extant genera it is at least 0.12–0.14.

In dorsal view, the compound eyes of *Chibiphemera* **gen. nov.** (as in recent *Nadinetella*) appear clearly fused; however, in lateral view, the two portions of the eyes in the fossil genus are only weakly separated, in contrast to extant genera, where the division between the upper and lower portions is more pronounced (Fig. 2A, C).

Clear differences are evident in the shape and venation of the forewings. The forewing of *Chibiphemera* **gen. nov.** is not narrow and possesses a jagged anterior margin; in *Lestagella* and *Lithogloea,* only the hind margin is jagged, whereas in the other extant genera both margins are smooth (Fig. 4A, B; Table 3). Compared to extant taxa, *C. cretalota* **sp. nov.** displays a more restricted distribution of the small free intercalaries (located in the iMP–A_2_ region only), accompanied by increased density of cross venation (e.g., between Sc and RA). The fossil taxon also exhibits unique proportions in the unbranched and branched sections of RS (0.14 in *C. cretalota* **sp. nov.**; 0.18–0.28 in extant species) and MP (0.40–0.42 and 0.20–0.37, respectively). Marked differences are also observed in the structure and venation of the cubital and anal sectors, particularly in the number of small free intercalaries and cross-veins (for detailed information, see Table 3).

Significant differences between *C. cretalota* **sp. nov.** and extant species are evident in the hind wings, which are strongly diminished in the fossil species and exhibit simplified venation (Fig. 4C, D; Table 3). Among all Teloganodidae, only *Chibiphemera* **gen. nov.** and the Malagasy *Manohyphella* possess costal projections on the hind wing that are clearly positioned distally. However, *C. cretalota* **sp. nov.** is uniquely characterised by a marked reduction of longitudinal and cross venation of the hind wings, the absence of vein triads and the MP fork, combined with the lack of free intercalaries and cubital venation (Fig. 4C, D; Table 3).

The fossil genus is characterised by a unique combination of pretarsal claw structure. The forelegs bear pad-like claws that are blunt apically, while the middle and hind legs possess claws that are hooked at the tip. Similar pad-like foreleg claws have been described in male imagines of *Lestagella* and *Nadinetella*. However, unlike *Chibiphemera* **gen. nov.**, the pretarsal claws of the middle and hind legs in these genera are always dissimilar, i.e. one claw is hooked while the other is blunt at the apex (Fig. 5B–D; Table 3).

When comparing the distribution of gill socket vestiges on the abdominal segments of the fossil genus and extant taxa, it is notable that in *Chibiphemera* **gen. nov.**, such sockets are located on terga II–V (the same in *Manohyphella* and *Nadinetella*), in contrast to *Ephemerellina*, *Lestagella*, and *Lithogloea*, where they are present on terga II–VI. It should also be noted that the adults of *Indoganodes* are currently unknown, and therefore, the condition of the gill socket vestiges in this genus remains uncertain. Thus, *Indoganodes* may also exhibit sockets on abdominal terga II–VI.

Essential differences between the taxa analysed here are observed in the structure of the male genitalia. While the penis lobes of all extant genera of Teloganodidae, including Afrotropical representatives, are almost fused, in *Chibiphemera* **gen. nov.**, the lobes are deeply separated. The shape of the penes in a fossil species is clearly distinguishable, consisting of stick-like lobes that are bent inward at the apex (Fig. 5A; Table 3). Similarly marked differences are evident in the structure of the styliger, especially in the proportions of the gonostyli segments. In the fossil *C. cretalota* **sp. nov.**, the first distal segment is the longest, i.e. more than twice the length of segment II, whereas in Afrotropical Teloganodidae, it is the second segment that is the longest (Fig. 5A; Table 3). A further relevant character to compare *Chibiphemera* **gen. nov.** with extant Pantricorythi is a median protuberance between the bases of the genital forceps, which is e.g. well developed in *Teloganella* and also present, though less pronounced, in *Ephemerythus* [31, 32, 100]. Because adults of *Indoganodes* have not yet been described, comparisons of male genitalia across all extant genera cannot be considered comprehensive.

***Bharataganodes*** **gen. nov.**

*Teloganella gurhaensis* Agnihotri et al., 2020 was established for a single compressed larva from the Palana Formation (Gurha lignite mine, Rajasthan, India) [29]. The fossil larva originally attributed to *Teloganella* Ulmer, 1939 is the third species described in this genus. Two extant representatives of *Teloganella* are currently distributed in Malaysia, Indonesia, and India [32]. Based on the original description and images [29], there is no certainty that *T. gurhaensis* can be assigned to the genus *Teloganella*, although the fossil taxon undoubtedly belongs to Pantricorythi (see below). While the placement of *Teloganella* within Pantricorythi (superfamily Ephemerelloidea Klapálek, 1909) appears well supported, its systematic position within the families assigned here is subject of debate [31, 32, 46, 107, 108]. Several authors assign *Teloganella* to the monogeneric family Teloganellidae McCafferty & Wang, 2000 [30, 42, 83]. Other researchers have included this genus within Melanemerellidae as subfamily Teloganellinae [98], to Ephemerellidae [109, 110], or to Tricorythidae [31, 111], and also to Teloganodidae [106, 112].

When determining the systematic position of the fossil larva from the Palana Formation (India), its fossilisation and preservation should be taken into account, as these may affect the interpretation of morphological characters. Although the larva is relatively well preserved, with its body almost complete except for the right hind leg, some important morphological structures are either not preserved, damaged, or difficult to interpret.

(i) The fossil specimen of *B. gurhaensis ***comb. nov.** is represented by a larva rather than a larval exuvia, based on the absence of visible cuticular dissections or ruptures on the dorsal side of the thorax, particularly on the mesonotum. The larva is visible only from the dorsal side, and thus, details of the mouthparts are not available. This precludes the possibility to analyse the shape of the labium in order to confirm the attribution of *B. gurhaensis ***comb. nov.** to the genus *Teloganella* within Teloganellidae, as determined in the original description [29]. Kluge et al. ([32]: p. 288, fig. 21) recognised a deep labial cleft between the glossae as an autapomorphy of *Teloganella*. The same condition was reported for the newly established monotypic genus *Janohyphella* Selvakumar et al., 2014 from southern India [25: p. 90, fig. 9], which was later synonymised with *Teloganella* [32]. Only remnants of the medially concave labrum and presumed traces of mandibular setation could be observed in the fossilised larva of *B. gurhaensis ***comb. nov.** (Fig. 6B).
(ii) It can be assumed that the larva of *B. gurhaensis ***comb. nov.** underwent partial deformation during fossilisation. While head, thorax, and legs were compressed without visible changes in proportions or symmetry, the abdominal segments appear slightly asymmetrical and stretched relative to the longitudinal body axis. As a result, the abdomen appears visually elongated, significantly longer than the thorax. Such longitudinal separation of abdominal segments during dorsoventral compression is quite common in fossil larvae. However, the length of the abdomen, calculated by measuring each segment individually, indicates that the actual proportion between the thorax and abdomen is different. According to [29], the lengths of the prothorax and mesothorax of *B. gurhaensis ***comb. nov.** are 2.00 mm, and the length of the abdomen is 3.40 mm. Thus, excluding the metathorax, the thorax/abdomen length ratio is 0.59. However, when measuring the length of each segment of the abdomen separately, and including the metathorax size in the analysis, the thorax/abdomen length ratio in the holotype of *B. gurhaensis ***comb. nov.** is approximately 0.68.

- Kluge et al. [32: p. 288] listed characteristic larval body proportions among the autapomorphies of *Teloganella*, namely the presence of a shortened thorax and an elongated abdomen (see also [31]: p. 325, fig. 1 [30]: p. 83, fig. 10 [25]: p. 90, fig. 2); the corresponding calculated thorax/abdomen length ratio based on published data is approximately 0.50. In all genera of Teloganodidae, this ratio is always more than 0.60 (*e.g*. in the genus *Indoganodes* with markedly elongated larval prothorax, the ratio is approximately 0.66 [25, 26]). Thus, the ratio specified for the extinct *Bharataganodes* **gen. nov.** is more similar to that in Teloganodidae than to *Teloganella*.
  On the other hand, it should be noted that many Teloganodidae are characterised by intraspecific variation in body shape during larval growth, as well as some differentiation between male and female larvae [40, 42]. The larval holotype of *B. gurhaensis* **comb. nov.** probably represents one of the last instars, judging by the degree of wing pad development. However, it is difficult to determine the sex of the larva with certainty, as it is visible from the dorsal side. At the same time, it cannot be excluded that the dark spots on the posterior part of the head of *B. gurhaensis* **comb. nov.** may represent traces of the dorsal portion of enlarged male compound eyes (see [29]: 139, fig. 2C).

(iii) Together with the head and the thorax, the legs are usually the most sclerotised part of the larval body, their shape being little altered by fossilisation. The legs of *B. gurhaensis* **comb. nov.** are of characteristic shape, with the forefemora greatly expanded and only slightly asymmetrical. The widest part of the forefemur in the fossil larva is close to the middle, with the inner margin more convex than the outer. The middle and hind femora are robust, broad, and clearly asymmetrical due to the more pronounced convexity of the outer margin, while the inner margin is more or less straight.

Similar to *Bharataganodes* **gen. nov.**, a comparable combination of features in the structure and shape of the forefemora is observed in the extant Malagasy genus *Manohyphella* within Teloganodidae. However, in *Manohyphella* the forefemora are more asymmetrical than in the fossil genus, due to the proximal location of its widest part ([86]: pp. 43–46, figs. 1, 8–10). A similar condition of this character is found in *L. penicillata* Barnard, 1940, within the monotypic South African genus *Lestagella*, whose larvae have the forefemora widest near the base [17, 40, 42].

In *Teloganella* (Teloganellidae), the legs exhibit characteristic proportions:

1. The forefemora are greatly widened and clearly asymmetrical, with the widest part positioned far distally ([31]: p. 325, figs. 1, 2; [25]: p. 90, fig. 10; [32]: p. 291, fig. 13);
2. The middle femora are also expanded, markedly asymmetrical, and widest near the distal end ([32]: fig. 14);
3. The hind femora are likewise widened and asymmetrical, but the convexity of their outer margin is less pronounced.
4. *Teloganella* also differs from *Bharataganodes ***gen. nov. **in the characteristic shape of the tibiae, which are widest in the proximal part, in contrast to *Bharataganodes ***gen. nov.**, which has the foretibiae expanded distally and the middle tibiae with parallel margins.

Thus, a detailed re-examination of the holotype of *B. gurhaensis* **comb. nov.** has enabled us to describe additional details of the larval morphology that were omitted in the original description [29]. Furthermore, it has become clear that the larva does not belong to the family Teloganellidae and cannot be associated with the genus *Teloganella*, to which this fossil taxon was initially assigned. As the original description of the fossil larva did not include a more detailed analysis of its systematic position, we present below several arguments in favour of placing *Bharataganodes* **gen. nov.** within the family Teloganodidae (Ephemerelloidea):

- The extinct taxon clearly is a pannote mayfly [30, 97], as the forewing pads are separated from each other by less than half of their length (Fig. 6A[29]: p. 139, fig. 2A, B).
- We exclude an assignment of the genus *Bharataganodes ***gen. nov.** to the superfamily Caenoidea Edmunds & Traver, 1954 (or Caenotergaliae Kluge, 2000), based on the structure and shape of larval foreprotoptera, which are fused to the mesonotum at their base and partly along the proximal part of the basitornal margin (in contrast to Caenoidea with foreprotoptera fused to the mesonotum with their basitornal and partly tornoapical margins [46]). The placement of the larval protoptera of the forewing inside the wing pads in Teloganodidae and Pantricorythi in general is well illustrated ([99]: p. 23, fig. 6 for Tricorythidae; for *Indoganodes* see [25]: p. 92, fig. 21 and [26]: p. 125, fig. 1B, E).
- Leptophlebioidea can clearly be excluded based on shapes of head and legs in the fossil larva, as well as the presence of a diminished, unforked and styliform gill I.
- The characters of the larva discussed in point (iii) and analysed in detail above, together with the presumed presence of a V-shaped impression on the pronotum, allow *Bharataganodes ***gen. nov.** to be assigned to the superfamily Ephemerelloidea. In addition to these characters, Kluge [46] also noted that the foreprotoptera of Ephemerelloidea are convergent at their apices. In the fossil larva, only the apex of the left foreprotopteron is preserved, and it is positioned approximately parallel to the body axis. However, it should be noted that the fossil larva was compressed during fossilisation, and the orientation of the protoptera may have been altered.
- Except for the left gill I, no other gill pairs have been preserved in *Bharataganodes ***gen. nov.**, which complicates the analysis of the systematic relationships between this extinct genus and the rest of Ephemerelloidea.

The assignment of *Bharataganodes* **gen. nov.** to Pantricorythi is supported by the well-developed, stout setation along both the inner and outer margins of all legs. In the proximal part of the fore- and middle femora of the fossil larva, there are robust setae of varying lengths, some of which evidently represent coxal setae, a condition characteristic of Teloganodidae [24, 46, 98]. On the other hand, despite its placement within Pantricorythi, the larva of *Bharataganodes* **gen. nov.** also exhibits several characters shared with larvae of the genus *Cincticostella* Allen, 1971, which belongs to the family Ephemerellidae. Among these features, the most notable is the presence of prothoracic anterolateral projections, although in *Cincticostella,* these are apically rounded. In some species of *Cincticostella*, similarities can also be observed in the shape of the robust claws and in the head, which is expanded anteriorly [46, 113].

Undoubtedly, the fossil larva does not belong to the taxon Tricoryptera Kluge, 2004, which was established for several families of Pannota mayflies (namely Ephemerythidae; Leptohyphidae; Machadorythidae; and Tricorythidae) from the Afrotropical and Oriental Regions and the Americas. Unlike in these families, the foreprotoptera of the fossil larva bear a well-defined tornus. Moreover, extant larvae of the families listed above differ markedly in their habitus from *Bharataganodes* **gen. nov.**, particularly such taxa as *Dicercomyzon* Demoulin, 1954 (Dicercomyzidae), *Machadorythus* Demoulin, 1959 (Machadorythidae), and most South American Leptohyphidae.

The family Vietnamellidae can be excluded based on the larval structure of the head and legs. In contrast to *Bharataganodes* **gen. nov.**, extant larvae of *Vietnamella* possess prominent, anteriorly directed projections on the head, in combination with forefemora that are distinctly widened proximally and bear a heavily denticulate inner margin [34, 46, 113–117]. Vietnamellidae are also known from fossils, but only as imagines [48, 95].

Similarly, *Bharataganodes* **gen. nov.** can be excluded from the family Austremerellidae, as the larvae of its sole extant genus, *Austremerella*, have lost the first pair of gills and possess paired submedian projections on the abdominal terga (the latter character is also shared with *Vietnamella*); the forefemora of *Austremerella* are distinctly asymmetrical, with an enlarged outer margin and a relatively straight inner margin [22, 30, 92].

Two lineages of Teloganodidae can be established based on larvae. The Oriental lineage is distinguished from the Afrotropical one by the absence of gills on abdominal segment I and by the reduction of the median caudal filament, which gives the nymphs a two-tailed appearance [17, 24, 30, 46]. Only the Oriental genus *Indoganodes* possesses a well-developed paracercus and, aside from this character, exhibits notable similarity to the South African genus *Ephemerellina* [25, 26]. *Manohyphella* is evidently more closely allied with the Afrotropical lineage than with the Oriental one. The presence of a well-developed terminal filament (as in *Teloganella*), and a patch of setae along the outer margin of the mandibles (also as in *Teloganella*, see [86]: pp. 44–45, figs. 3, 4 and [25]: p. 90, figs. 6–7), are two characters shared among all Afrotropical genera, including *Manohyphella*.

Judging by these characteristics, *Bharataganodes* **gen. nov.** appears more closely related to the Afrotropical genera of Teloganodidae than to the Oriental taxa, because the fossil larva possesses three caudal filaments and remnants of the gill I shaped similarly to Teloganellidae (for comparison see Table 4). The presumed remnants of relatively dense mandibular setation preserved in *Bharataganodes* **gen. nov.** can also suggest an affinity with African and Malagasy Teloganodidae, which exhibit similar setation along the outer margin of the mandibles (Fig. 6B; see also [24, 40, 86]), in contrast to Oriental species, which typically bear only a single thin or stout seta in the same position [24, 27, 28, 118]. Notably, a comparable patch of setae was described and illustrated for *Teloganella* by Selvakumar et al. ([25]: p. 90, figs. 6, 7).

In their revision of the family Teloganodidae, McCafferty & Wang [22] noted that in Afrotropical genera “the forefemora are characteristically flattened, with stout setae arranged in a transverse row”. In *B. gurhaensis* **comb. nov.,** this row is not preserved, but the forefemora are distinctly flattened. This feature was supported by subsequent studies, which reaffirmed its presence in Afrotropical genera [24, 40].

In this way, *Bharataganodes* **gen. nov.** shares all distinguishing characteristics with Afrotropical genera, namely *Ephemerellina*, *Lestagella*, *Lithogloea*, *Nadinetella*, and *Manohyphella* (Table 4).

Despite its evident similarity to *Lestagella* and *Manohyphella* in body setation and with several shared features in foreleg morphology, particularly the markedly enlarged and distinctly asymmetrical femora, *Bharataganodes* **gen. nov.** exhibits a suite of characters that clearly distinguish it from other Afrotropical Teloganodidae (Table 4): (**i**) the larval head, although covered with a dense fringe of long, stout setae, remains prognathous, broadly rounded, and anteriorly irregularly rectangular; (**ii**) the pronotum exhibits a distinctive, conspicuous anterolateral angle that is protruded and apically pointed; (**iii**) the foretibiae of *B. gurhaensis* **comb. nov.** are extremely short, widest distally, and distinctly shorter than the tarsi; (**iv**) the foreclaws are robust and hooked, bearing a distinct proximal tubercle but lacking any trace of preserved dentition; (**v**) the abdominal terga lack any discernible submedian projections, while the posterolateral projections on the terminal segments are markedly larger and more robust than in most African genera, *Manohyphella* and *Lithogloea* possibly excepted.

Analysing the general body shape, the fossil larva appears closely related to *Lestagella*, and particularly to *Manohyphella*, with which it probably shares certain characteristics pertaining to ecological and habitat preferences (see below). On the other hand, as demonstrated for *Lestagella* [35] and for *Manohyphella* [42], the larval body shape exhibits some variation between sexes and across developmental stages.

#### Phylogenetic relationships of *Chibiphemera***gen. nov.** and *Bharataganodes* gen. nov.

Teloganodidae have previously been treated as a subfamily within Ephemerellidae [106]. They have also been assigned to the taxon Pantricorythi, which includes all Ephemerelloidea except Ephemerellidae sensu stricto [46]. Likewise, the genus *Teloganella* was alternatively placed in the families Ephemerellidae, Tricorythidae, Teloganodidae, and Melanemerellidae, until it was finally assigned to the monogeneric family Teloganellidae [32].

Earlier attempts to reconstruct the phylogenetic relationships within Teloganodidae were based on cladistic analyses of larval characters, as well as egg and adult morphological features. McCafferty & Wang [22] proposed a cladogram for the genera they included within Teloganodidae, which also encompassed *Vietnamella* and *Austremerella* (see also above). In this tree, the clade comprising *Vietnamella* and *Austremerella* is sister to the remaining Teloganodidae, which includes both African and Oriental lineages. However, *Manohyphella* was not included, as at that time it was considered to belong to the family Tricorythidae [22, 31]. A short time later, McCafferty & Wang [30] proposed a cladogram for the major lineages of Pannota. This included Teloganodidae, which were recovered as the sister group to the remaining Pantricorythi, with the *Austremerella* group sensu McCafferty & Wang (2000) (i.e. *Austremerella* and *Vietnamella*) representing the most basal lineage. The changes in the systematic position of the genus *Vietnamella* and Vietnamellidae were discussed in detail by Godunko et al. [48, 95], who included Cretaceous representatives into Vietnamellidae.

McCafferty & Benstead [86] showed that *Manohyphella* represents the sister lineage to the African and Oriental Teloganodidae, with the exception of *Ephemerellina*, which was resolved as the most basal clade. Using a dataset of 31 characters, Molineri & Domínguez [85] proposed a cladogram for several lineages within Ephemerelloidea, with particular emphasis on the position of *Melanemerella*. The cladogram clearly illustrates the complex and often ambiguous relationships among the families comprising this superfamily. The African *Teloganodidae*, represented by the genera *Lithogloea* and *Ephemerellina*, were considered as separate basal lineages to the remaining Pantricorythi. However, *Ephemerythus*, which occurs in tropical regions of South Africa and morphologically closely resembles Teloganodidae, nested within the *Austremerella* clade [85].

The clade Manohyphella + (Teloganella + Melanemerella) is also noteworthy, as Teloganella and Melanemerella each represent a separate monotypic family, whereas Manohyphella is a genus within Teloganodidae. Together, these three genera are distributed across three different biogeographical realms.

Jacobus & McCafferty [98] refined the phylogeny of Pannota by incorporating teloganodid genera, including new data on the diversity, distribution, and systematics of Ephemerelloidea, together with the description of additional characters. Their revision was based on an analysis of 46 characters across 34 OTUs and resulted in several significant changes. Most notably, the monotypic genus *Philolimnias* Hong, 1979, was placed in its own monogeneric family, Philolimniidae Jacobus & McCafferty, 2006, proposed as the sister group to the remaining Pantricorythi (but see also [94]). The family Vietnamellidae was restricted to the genus *Vietnamella*, whilst the family Austremerellidae was reinstated for the Australian genus *Austremerella* (see also [48]). Additionally, Jacobus & McCafferty [98] included *Teloganella* within the family Melanemerellidae.

While Ogden et al. [108] presented a phylogenetic tree for the subfamily Ephemerellinae (Ephemerellidae) based on combined molecular and morphological data, comparable studies incorporating the majority of genera within Pantricorythi are still lacking. Subsequent analyses using similar combined methodologies have supported the monophyly of Pantricorythi [107, 119]. However, the Malagasy genus *Manohyphella* has been shown to be sister to a clade comprising the Oriental genus *Derlethina* (Teloganodidae) and a common lineage of the African Tricorythidae and Leptohyphidae (see [108]). These findings suggest that the family Teloganodidae, comprising *Manohyphella*, constitutes a paraphyletic taxon. It should be noted, however, that while *Derlethina* was included in the analysis, the African genera of Teloganodidae were not represented.

The generic affinities within Teloganodidae, as well as their phylogenetic relationships with closely related families, remain uncertain [17, 27, 46]. Pereira-da-Conceicao [17] highlighted the speculative nature of certain phylogenetic reconstructions and the conclusions derived from them, primarily due to the absence of most Oriental Teloganodidae, in addition to the limited number of specimens representing Melanemerellidae and Ephemerythidae. Based on the results of a combined analysis of the mitochondrial genes COI, 16S, and 12S, she considered the possibility of downranking Ephemerythidae and Melanemerellidae to the level of subfamilies within Teloganodidae, and of establishing a separate subfamily for the Oriental lineage of teloganodid genera. Nevertheless, the author emphasised the need for more detailed studies and a more comprehensively sampled dataset [17].

The phylogenetic analysis ([17]: p. 68, fig. 4.3) also revealed the paraphyly of Teloganodidae, with Ephemerythidae + Melanemerellidae forming the sistergroup to the African genera, altogether forming the sistergroup to Asian Teloganodidae. Within this framework, the Oriental species *Dudgeodes ulmeri* occupies a basal position as the sister group to the remainder of the clade. While noting the insufficiency of available data for a robust phylogenetic reconstruction within Afrotropical Teloganodidae, Pereira-da-Conceicao [17] also reported a well-supported relationship between the Madagascan genus *Manohyphella* and the remainder of the South African Teloganodidae, a relationship apparently closer than with the Asian lineage. This contrasts with the cladistic analysis of McCafferty & Benstead ([86]: p. 47, fig. 14), in which *Manohyphella* was placed as the basal branch of the entire Teloganodidae clade (excluding *Ephemerellina*). In the analysis by Pereira-da-Conceicao [17], *Manohyphella* is instead positioned closer to the ‘non-fringed’ South African genera, i.e. a group whose larvae lack the long setae on the head. This group, comprising *Lithogloea*, *Nadinetella*, and *Ephemerellina*, and considered to form a separate ‘non-fringed’ clade in the phylogenetic tree proposed. Conversely, the ‘fringed’ clade, represented by *Lestagella*, was positioned as a basal branch that includes all other Afrotropical Teloganodidae (i.e. the ‘non-fringed’ genera + *Manohyphella*). Thus, these results support a single origin of the Afrotropical Teloganodidae, which aligns with earlier biogeographical and cladistic studies proposing a Gondwanan origin for the family [19, 20, 22–25].

A more detailed evaluation of existing phylogenetic hypotheses and perspectives concerning relationships within Teloganodidae, including the connection between African and Asian lineages, is essential to clarify the potential placement of new fossil taxa within the family. Given that the two extinct genera are each known only from a single male imago and a single larva, respectively, the establishment of their phylogenetic affinities within Teloganodidae remains problematic.

The fossilised larva, although represented by a specimen exhibiting a complete and unbroken body, has lost its gill plates, except the presumed remains of the left first gill. Owing to the visibility of the body in dorsal aspect, it is not possible to describe or compare the morphology of the mouthparts and prosternum with those of extant taxa. Our conclusions are necessarily based on a limited set of characters. Nevertheless, these are sufficient to support the confident assertion that *Bharataganodes* **gen. nov.**, from the Palana Formation, is most closely allied with Afrotropical genera of Teloganodidae (see above). Certain aspects of leg morphology in the fossil larva, particularly the markedly flattened, greatly expanded and asymmetrical forelegs, as well as the dense setation of the body, especially along the anterior and lateral margins of the head, show strong affinities with *Lestagella* and *Manohyphella*. These features suggest, despite the paucity of other diagnostic morphological characters, that all these genera are closely related.

Although some discrepancies persist in the interpretation of the phylogenetic positions of extant *Lestagella* and *Manohyphella*, both genera are frequently recovered as basal lineages within the Afrotropical Teloganodidae in many phylogenetic reconstructions. Therefore, if our hypothesis regarding the close relationship of *Bharataganodes* **gen. nov.** to *Lestagella* and *Manohyphella* is correct, the extinct taxon may either represent a sister lineage to one of these extant genera or occupy an even more basal position within the Afrotropical clade. However, given the unresolved phylogenetic relationships both within the African and Malagasy Teloganodidae, and between Afrotropical and Oriental genera, coupled with the limited palaeontological evidence currently available, we approach this inference with due caution.

The specimen of *Chibiphemera* **gen. nov.** is well preserved, permitting the description of several important diagnostic characters. Although *Chibiphemera cretalota* **sp. nov.** exhibits apomorphic features in the hind wing, most notably a marked reduction in size and venation, and a costal process that is strongly displaced distally (Fig. 4C, D; Table 3), the male imago retains plesiomorphic characters in the structure of the forewings and genitalia. The forewings display a well-developed network of transversal venation, accompanied by a reduction in the number and distribution of small free intercalaries (Fig. 4A, B). In contrast to the specialised genitalia of most Pantricorythi, the genitalia of *Chibiphemera* **gen. nov.** are more generalised, as typical of many mayfly lineages: the penis lobes are deeply separated and stick-like, and the gonostyli are four-segmented, with the first distal segment being the longest and the terminal segment the shortest.

Male genitalia similar to those of *Chibiphemera* **gen. nov.** are observed in *Burmella* Godunko et al., 2021, a Cretaceous mayfly genus in Burmese amber assigned to the family Vietnamellidae within Ephemerelloidea (see also [95]: p. 109, fig. 6C, D [48]: p. 26, fig. 8A–C). In contrast to the extant genus *Vietnamella* (Vietnamellidae), whose males exhibit genitalia characteristics of Pantricorythi with medially almost entirely fused penes, *Burmella* possesses largely separated penis lobes, thereby retaining a plesiomorphic condition. Additionally, *Burmella* exhibits a markedly elongate first segment of gonostyli. Vietnamellidae, along with the closely related Australian monogeneric family Austremerellidae, are generally regarded to represent the most basal lineages of Pantricorythi. As with *Bharataganodes* **gen. nov.**, the absence of other life stages for *Chibiphemera* **gen. nov.** limits a comprehensive phylogenetic interpretation. Nevertheless, the plesiomorphies discovered in this Mesozoic taxon allow for the cautious suggestion that it may occupy a basal position relative to other representatives of Teloganodidae.

#### Palaeobiogeographical considerations

The prevailing consensus regarding the evolutionary history of the Teloganodidae is that they represent an ancient Gondwanan relict, whose distribution was shaped primarily by tectonic vicariance. The family dispersal is thought to have been driven by the breakup of Gondwana and the subsequent drift of its constituent landmasses across the Tethys [17, 19, 20, 22, 24–26, 98].

To define criteria for identifying taxa of Gondwanan origin whose present distributions are attributable to vicariance, Datta-Roy & Karanth [61] proposed that (a) a group must be monophyletic and occur across former Gondwanan fragments; (b) phylogenetic divergence of taxa should correspond to geological events associated with Gondwana breakup; and (c) estimated divergence times must align with periods of landmass separation. Based on these criteria and our data, we conclude that Teloganodidae originated in Gondwana and that the distribution of several of its lineages was shaped exclusively by the fragmentation of this supercontinent.

The assumption that Teloganodidae originated in southern Pangaea around 200 Ma is based on the inclusion of the Australian genus *Austremerella* within the family [22]. The Afrotropical lineage is considered the oldest among extant Teloganodidae (see also [17]). This dating would also imply a correspondingly earlier origin for Ephemerelloidea as a whole, potentially extending their origin back to the Palaeozoic [30].

By contrast, following Pescador et al. [120], we assume that the vicariant split between Ephemerellidae and Pantricorythi occurred during the Jurassic and coincided with the breakup of Pangaea (see [48] for details). Before this event, the two ancestral lineages most likely dispersed and diversified across several modern continents no later than the early Jurassic (200–175 Ma). This interpretation suggests a more recent origin of Ephemerelloidea than suggested by McCafferty & Wang [22, 30], placing it in the earliest Mesozoic, possibly following the P–Tr extinction.

Evidence from geographically disparate Mesozoic and Cenozoic deposits in Europe, Asia, South America, and Australia underscores the pivotal role of vicariance linked to Pangaean fragmentation in the evolution and diversification of several families, whose origins are more ancient than previously thought [47–49]. Nevertheless, no evidence supports the presence of extant Ephemeroptera superfamilies or families in the Palaeozoic, before the P–Tr extinction [1, 121]. In contrast, Plecoptera appear to have originated earlier, with divergence times extending into the Late Palaeozoic [122–126].

Using Reconstruct Ancestral State in Phylogenies (RASP) analyses, Pereira-da-Conceicao proposed a Pangaean origin for a clade including Teloganodidae, Ephemerythidae, and Melanemerellidae, with an earlier widespread distribution across that supercontinent [17]. This scenario appears valid for all Pantricorythi, or the ‘Gondwanan clade’ of Ephemerelloidea [30]. The early divergence of early diverged families such as Vietnamellidae and Austremerellidae supports this interpretation, aligning with the palaeogeography of East Gondwana. Dispersal of Austremerellidae eastward was likely completed by approximately 133–130 Ma, coinciding with the onset of East Gondwana’s breakup and the north-westward drift of the circum-Indian blocks and microcontinents from the Australia–Antarctica margin [127–131]. Their divergence from other Pantricorythi may have occurred early during the Middle–Late Jurassic [48]. Under this scenario, the easternmost Austremerellidae became isolated in Australia, while Vietnamellidae diversified in isolation as a result of the WBT drift.

Pereira-da-Conceicao also highlighted the role of Gondwana’s ‘mega-desert’, an extensive hyper-arid belt spanning South America, Antarctica, and Madagascar, during the Middle–Early Jurassic [17]. This desert acted as a significant biogeographic barrier, separating the northern (Ephemerythidae + Melanemerellidae) and southern (Teloganodidae) lineages of Pantricorythi. She suggested that ancestors of Teloganodidae circumvented this barrier and dispersed northeastward ([17]: fig. 5.7b). This desert is considered a key palaeoenvironmental feature of the Early Mesozoic, profoundly shaping terrestrial ecosystems by limiting dispersal and influencing evolutionary trajectories [132, 133]. This may explain the early divergence (c. 160–150 Ma) of the Oriental Teloganodidae lineage, although molecular phylogenetic analyses provide only weak support for this scenario ([17]: figs. 5.2, 5.7c, d).

A shorter dispersal route from southern Africa to India and Madagascar, before their separation from the rest of Gondwana, is also plausible [17]. Climatic arid conditions in southern Africa close to the end of the Jurassic and the beginning of the Cretaceous [134, 135] likely acted as an additional dispersal barrier, preventing the dispersal of aquatic insects and promoting the divergence and isolation of African and Asian Teloganodidae [17]. Evidence from the Clarens Formation (Karoo Basin, South Africa) confirms prolonged arid, windy conditions punctuated by wet-dry cycles, with intermittent fluvial and lacustrine habitats persisting among dunes of the Karoo sand sea during the Sinemurian–Pliensbachian in Early Jurassic between ~190–175 Ma [136]. Thus, even for these harsh climatic conditions, which included alternating wet and dry climatic cycles, the presence of various fluvial and lacustrine settings during Karoo sand sea existence was established [137–140]. Whether these ephemeral freshwater ecosystems could have served as refuges for rheophilic stenobiotic Teloganodidae during their dispersal from South Africa to the northeast toward the Indian Plate remains uncertain. Fossil Ephemeroptera and other aquatic insects with similar lifestyles (e.g., Plecoptera) from this region remain extremely scarce [124, 126].

By the mid-Jurassic (c. 170–150 Ma), biotic exchange between the landmasses of Gondwana was still possible, despite the onset of rifting between Lemuria and Africa (~160–158 Ma) [17, 48, 63, 141]. Migration remained feasible in East Gondwana until at least 140–135 Ma, facilitating interchange between South Africa, Lemuria, Australia, and Antarctica [18, 131, 142]. Climatic amelioration (i.e., more humid and temperate climate) at the onset of the Cretaceous (c. 140 Ma), linked to the opening of the Indian Ocean, likely enabled some Teloganodidae ancestors (including those of *Chibiphemera ***gen. nov.** and *Bharataganodes* **gen. nov.**) to traverse the arid barriers and disperse eastward and northeastward, including to WBT, either via the most direct route while it was still incorporated to Gondwana, or through a more circuitous path around Antarctica, along the eastern margin of the desert zone.

In one way or another, prior to the separation of the Indian subcontinent and associated terranes from Gondwana, ancestral representatives of several Teloganodidae lineages were widely distributed across the region (Fig. 9).

(i) The ancestors of *Manohyphella* likely colonised Madagascar around 140 Ma. In response to the harsh, arid conditions prevailing in the lowlands, they adapted to isolated mountain waterholes within humid environments, resulting in long-term geographic and ecological isolation. Having successfully persisted through multiple glacial and interglacial cycles [17], *Manohyphella* appears to have undergone limited morphological or ecological diversification, in contrast to more dynamic families such as Heptageniidae Needham, 1901 [90]. It thus represents an example of evolutionary stasis over tens of millions of years.
(ii) Substantial volcanic activity in Africa during the mid-Cretaceous (~110 Ma) probably reinforced the isolation of the South African Teloganodidae lineage. Simultaneously, tectonic processes linked to the breakup of Gondwana curtailed contact from both the east and west. These combined barriers likely stimulated diversification within the African lineage [17].
(iii) *Chibiphemera ***gen. nov.**, described from Burmese amber, is the oldest known member of Teloganodidae and retains adult plesiomorphies indicative of a position of early diverging genus within the family. The timing and pathway of the separation of the Burmese Block from eastern Gondwana remain debated, as does its precise location in the Tethys at the time of the Burmese amber deposition during northward migration [54, 143]. These uncertainties are crucial for reconstructing the biogeographic history of Asian faunal elements. The Burma Terrane may have acted as a “biotic ferry” for Gondwanan Pantricorythi, such as Vietnamellidae, while the amber biota includes both Gondwanan and also Laurasian taxa [48], suggesting that the Terrane’s position permitted interchange with Laurasia, particularly for passive or weak-flying insects such as mayflies. Models positioning the separation of the Burmese Block from Australia in the Late Jurassic and its collision with Southeast Asia during the Early Cretaceous [48, 53, 56, 144–147] remain consistent with this interpretation. Under such a scenario, early diverging lineages of both Pantricorythi (Vietnamellidae and Austremerellidae) and Teloganodidae (e.g., *Chibiphemera ***gen. nov.**) may have dispersed toward WBT while it was still Gondwanan via short or extended dispersal routes (Fig. 9). The West Burma Block is thought to have rifted from Gondwana during the Late Jurassic (c. 160–158 Ma), together with the Indian Subcontinent as one of its associated crustal fragments. Following its separation from India, possibly as an independent intra-oceanic arc or peri-Asian microplate before or shortly after 100 Ma [146–151], the block migrated northwards across the Tethys Ocean, carrying with it elements of Gondwanan biota. The eventual collision with the Asian margin is estimated to have taken place between 100 and 90 Ma, or perhaps slightly later, during the Santonian to Campanian stages (86.3–83.6 Ma) [152, 153]. However, several authors have proposed a more protracted timescale, suggesting that final accretion to Asia may not have occurred until the early Eocene [18, 154].

(iv) Jacobus & McCaffery suggested that the Oriental Teloganodidae and the Madagascan genus *Manohyphella* are sister groups [98], whereas Pereira-da-Conceicao argued for a much earlier divergence of the Asian lineage [17]. Sartori et al. hypothesised that the common ancestor of *Manohyphella* and the Oriental taxa became isolated during the breakup of Gondwana, evolving subsequently on a tectonic plate that included both Madagascar and the Indian Subcontinent [24]. Our findings support and extend this scenario by demonstrating that several Teloganodidae lineages, with evolutionary histories tracing back at least to the Early Cretaceous, may have dispersed from Africa to the Indian Subcontinent. These lineages initially evolved together for approximately 45–55 Ma before being separated by tectonic events within the Subcontinent. Following the rifting of WBT (c. 110–100 Ma), which acted as a ‘biotic ferry’ for *Chibiphemera ***gen. nov.**, Madagascar (with ancestor of *Manohyphella*) separated from the Indian landmass around 96–84 Ma [141].
(v) The Indian Plate, together with Greater India and Sri Lanka, acted as another, even more significant ‘biotic ferry’ under the ‘Out-of-India’ hypothesis [61], facilitating dispersal of Gondwanan biota into Asia. Ancestral lineages of the Oriental Teloganodidae and *Teloganella*, and the fossil *Bharataganodes ***gen. nov.** crossed the Tethys with the drifting plate during the Mesozoic and Cenozoic.

The first mayflies from Cambay amber [63] shed light on this process. Their analysis highlights the complexity and debated chronology of India’s northward drift, with multiple dispersal events shaping Asian faunal assemblages. Despite the mass extinction linked to Deccan Traps volcanism (peak ca. 66.9 ± 0.2 Ma [155]), many biotic elements survived in refugia such as the Palana Formation (57–54 Ma [156]). Cambay amber (54.5 Ma [64]), closer to the Deccan volcanic zone, preserves diverse and well-preserved assemblages, suggesting ecological recovery and renewed faunal exchange likely with Asia and Africa. However, many Gondwanan taxa had already gone extinct before India’s collision with Asia, leaving vacant niches that were subsequently colonized by Laurasia and African taxa via temporary land bridges or archipelagos [60, 61, 141, 157, 158]. As a result, multiple faunal affinities between the Cambay amber fauna and both Laurasian and African biota have been proposed [63].

Gondwana mayflies that drifted with India include *Bharataganodes* **gen. nov.** (Palana Formation) and *Aikahika* Sroka et al., 2025 (Cambay amber) [63]. The tectonic drift of India also shaped the distribution of Tricorythidae, recorded from Africa, Madagascar, India, Sri Lanka, and Southeast Asia [99, 159, 160]. Additional evidence for later colonisation from Africa during the Eocene comes from *Tricorythus* (s. str.) *meenakshi* in southern India, a genus otherwise known only from equatorial and southern Africa [102]. Similarly, Afro-Oriental genera such as *Povilla* Navás, 1912 and *Languidipes* Hubbard, 1984 (Asthenopodinae: Polymitarcyidae) mirror the biogeographical patterns of Teloganodidae [161].

In contrast to the Gondwanan genera and families discussed above, several other groups of Ephemeroptera may have colonised East Africa and Asia Minor much later, during pluvial periods of the Miocene, approximately 17 Ma [17]. McCafferty & Gillies [162] hypothesised such a scenario for the Ephemerid genus *Afromera* Demoulin, 1955. More recently, the first occurrence of *Cheleocloeon* (Baetidae) in India was reported, a genus otherwise diverse in Africa and Madagascar, but also known from the Arabian Peninsula [160, 163] (Kluge, 2016; Sivaruban et al., 2022).

Within the evolutionary history of Teloganodidae, the events following the collision of the Indian Plate with Asia (ca. 55–42 Ma) were briefly summarised by Sartori et al. [24]. According to their account, the genus *Teloganodes* followed two principal evolutionary trajectories: one lineage (*Teloganodes sensu stricto*) remained restricted to its original distribution range, while its sister lineage diversified and dispersed across Southeast Asia, giving rise to the extant genus *Dudgeodes* and to *Derlethina*, the most morphologically specialised and evolutionarily derived lineage. However, Selvakumar et al. [25] suggested that *Derlethina* may also have remained in India, thereby exhibiting an evolutionary and biogeographical pattern more closely aligned with that of *Teloganodes* and *Dudgeodes*.

#### Palaeoecological considerations

Studying fossil Vietnamellidae preserved in Burmese amber, Godunko et al. [48, 95] outlined the ecological key conditions required to support taxonomically diverse Ephemeroptera communities. The palaeohabitats of the Burmese mayfly fauna were most likely humid tropical forests with dense networks of watercourses. Pantricorythi were represented by the genera *Burmella* and *Chibiphemera* **gen. nov.**, which, together with other taxa possessing rheophilic larvae (e.g., Baetidae, Heptageniidae), probably inhabited specific stream and river segments within the rhithral zone.

The Palana Formation was deposited under a predominantly tropical, warm, and humid climate typical of the Early Eocene Climatic Optimum, a period marked by the expansion of modern biodiversity [164, 165]. Recent work by Kumar et al. [166] suggests a possible link between its deposition and a global hyperthermal event in the Late Palaeocene, driven by elevated atmospheric CO₂ levels and intensified precipitation. Palynological and palaeontological data further indicate the presence of rich tropical flora characteristic of the Palaeocene–Eocene transition [167, 168]. Collectively, this evidence suggests that the Palana Formation reflects a tropical rainforest ecosystem, or vegetation structurally and compositionally analogous to modern equatorial forests [168–170]. Such a humid climatic regime would also have facilitated the development of a complex mosaic of freshwater habitats, sustaining a diverse assemblage of both vertebrate and invertebrate aquatic taxa [29, 77, 79–81]. These findings indicate that the freshwater ecological parameters and climatic conditions of the Palana Formation were broadly comparable to those supporting extant Teloganodidae in lotic ecosystems within the tropical rainforests of South India, Sri Lanka, and Southeast Asia.

## Conclusions

This study provides new insights into the taxonomy, early evolutionary history, and palaeobiogeography of Teloganodidae, based on newly discovered and reinterpreted fossil material from mid-Cretaceous Burmese amber and Early Eocene deposits of western India. The establishment of two new genera, namely *Chibiphemera* **gen. nov.** based on the description of a male imago, and *Bharataganodes* **gen. nov.** based on a revised interpretation of the larval holotype of *Teloganella gurhaensis,* significantly enhances our understanding of the Late Mesozoic and Early Cenozoic evolution of this family.

The detachment of the West Burma Terrane from the Indian Plate during the mid-Cretaceous (c. 110–100 Ma) likely facilitated the eastward dispersal of the ancestor of *Chibiphemera* **gen. nov.**, acting as a ‘biotic ferry’. Similarly, the separation of Madagascar between ca. 96–84 Ma likely isolated the ancestor of *Manohyphella*, while ancestors of Oriental Teloganodidae, *Bharataganodes* **gen. nov.** and *Teloganella* (Teloganellidae) remained on the Indian Plate during its northward drift toward Asia.

Our results suggest two independent dispersal routes of Teloganodidae into Asia: (1) the earliest known lineage *Chibiphemera* **gen. nov.** dispersed with the Burma Terrane during the Cretaceous; and (2) a younger lineage, represented by *Bharataganodes* **gen. nov.**, and the ancestors of extant Oriental taxa, reached Asia via the Indian Plate during its northward migration. This study highlights the pivotal role of tectonic movements in shaping the early diversification and present-day distribution of mayflies, and underscores the palaeobiological significance of Indian and Myanmar fossil records in reconstructing dispersal pathways of Gondwanan biota.

## Materials and methods

### Type material

The present contribution is based on two fossil specimens. The holotype of *Chibiphemera cretalota* **gen. & sp. nov.** (male imago) is embedded in mid-Cretaceous Burmese amber discovered in a mine situated in the Hukawng Valley in Kachin State of Northern Myanmar. The Hukawng amber can be assigned to the middle Cretaceous (Upper Albian-Lower Cenomanian), with a maximum age of 98.79 ± 0.62 Ma as stated based on U-Pb zircon dating [143], which is equivalent to the earliest Cenomanian [91]. The holotype is currently deposited in the collection of Zhendong Lian (Tainan City, Taiwan, China) under inventory number T25L07001, and is available to researchers upon reasonable request. The holotype will be transferred to a new exhibition hall of the ZDL private museum, which will be publicly accessible, and the fossil collection will then be available for researchers.

The second fossil is a larval holotype specimen originally described as *Teloganella gurhaensis* from the Gurha lignite mine of Bikaner, Rajasthan, Northwest India [29]. This mine belongs to the Palana Formation. The age of the Palana Formation is disputable (see above); however stated as late Palaeocene–early Eocene in the original description [29], i.e. approximately 57–54 Ma [76]. The holotype of *Teloganella gurhaensis* is housed in the Museum of the Birbal Sahni Institute of Palaeosciences, Lucknow, India; BSIP specimen no. 41,813. Because it was not possible to loan the holotype for the present study, the original photographs of the larva were kindly provided by Dr Anumeha Shukla (Birbal Sahni Institute of Palaeosciences).

### Comparative material

Comparative material of extant Teloganodidae from the Afrotropical and Oriental Realms (larvae and adults) were studied using collections housed in the Institute of Entomology, BC CAS (České Budějovice, Czech Republic) and in the State Museum of Natural History Stuttgart (Stuttgart, Germany). Most of the comparative material of extant Teloganodidae from India is housed in the National Museum of Natural History collection NASU (Kyiv, Ukraine).

### Specimen processing and imaging

The methods used to examine and photograph the larval holotype of *Teloganella gurhaensis* are listed in Agnihotri et al. [29]. Photographs of *Chibiphemera cretalota* **sp. nov.** were taken under incident light using a Nikon SMZ1270 stereo microscope with the application Capture V2.2. Photo stacks were processed with Helicon Focus Pro 8.3.0 to obtain combined photographs with an extended depth of the field. All photographs were sharpened and adjusted in contrast and tonality in Adobe Photoshop™ (Adobe Systems Incorporated, San Jose, CA, USA). Line drawings were made using the clearest and sharpest images to visualise distinguishing morphological characters. Obtained images and photographs were processed using graphic tools embedded in Windows 10 and 11.

Comparative material of extant Teloganodidae from the Afrotropical and Oriental Realms (larvae and adults) was studied under a Leica M205 C (Leica Corporation, Wetzlar, Germany) and an Olympus SZX7 (Olympus Corporation, Tokyo, Japan) stereomicroscope. A detailed description of the optical equipment and methods used for photographing and processing the Indian material is provided by Martynov et al. [93].

### Terminology and taxonomic notes

The general anatomical morphology of adults is based on [16, 27, 46]. Abbreviations for wing veins follow [16, 17, 27, 28, 46, 48, 49, 94, 95]. Morphological terms and abbreviations of adult thorax morphology are adapted from [27, 28, 46, 171, 172].

### Nomenclatural acts

The electronic edition of this article conforms to the requirements of the amended International Code of Zoological Nomenclature, and hence the new names contained herein are available under that Code from the electronic edition of this article [88]. This published work and the nomenclatural acts it contains have been registered in ZooBank, the online registration system for the ICZN. The ZooBank LSIDs (Life Science Identifiers) can be resolved, and the associated information viewed through any standard web browser by appending the LSID to the prefix ‘‘http://zoobank.org/’’. The ZooBank LSID for this publication is: urn:lsid:zoobank.org:pub:7A23F28E-6510-45C9-81DF-C6ADB8C52CF8.

### New taxa registration

The following information was supplied regarding the registration of a newly described species:

Publication LSID: urn:lsid:zoobank.org:pub:7A23F28E-6510-45C9-81DF-C6ADB8C52CF8;

*Chibiphemera* **gen. nov.** LSID: urn:lsid:zoobank.org:act:04A0011C-1186-4AE7–B141-768913804A5D;

*Chibiphemera cretalota* **sp. nov.** LSID: urn:lsid:zoobank.org:act:090A9F26–A0C0-4D5F-BAE5-5E8722068DF2;

*Bharataganodes* **gen. nov.** LSID: urn:lsid:zoobank.org:act:5E8108B8-6193-4B89–AB3B-99C30D9E6F0B;

*Bharataganodes gurhaensis* **comb. nov.** LSID: urn:lsid:zoobank.org:act:F5CFE110–D935-43F6–A18D-49F381E8E8D1.

## Data Availability

The authors declare that all data supporting the findings of this study are available within the article. The holotype of *Chibiphemera cretalota* **sp. nov.** is presently stored in the Zhendong Lian collection (Tainan City, Taiwan, China) under inventory number T25L07001, until it will be transferred to the ZDL private museum. The holotype is available to researchers upon reasonable request. The holotype of *Bharataganodes gurhaensis* (Agnihotri, Chandra, Shukla, Singh & Mehrotra, 2020) **comb. nov.** is stored in the Museum of the Birbal Sahni Institute of Palaeosciences (Lucknow, India) under the inventory number 41,813. Requests for access to the fossil material of *Teloganella gurhaensis* should be addressed to the curator of the Palana Formation collection housed in the Birbal Sahni Institute of Palaeosciences, Lucknow, India. Comparative material of extant taxa of Teloganodidae is deposited in public research institutions (IE BC CAS, SMNS) and is available on request to the curators of these collections.
